# Exome-wide analysis of bi-allelic alterations identifies a Lynch phenotype in The Cancer Genome Atlas

**DOI:** 10.1186/s13073-018-0579-5

**Published:** 2018-09-14

**Authors:** Alexandra R. Buckley, Trey Ideker, Hannah Carter, Olivier Harismendy, Nicholas J. Schork

**Affiliations:** 1 Biomedical Sciences Graduate Program, University of California San Diego, La Jolla, CA USA; 2 Human Biology Program, J. Craig Venter Institute, La Jolla, CA USA; 3 Division of Medical Genetics, Department of Medicine, University of California San Diego, La Jolla, CA USA; 4 Moores Cancer Center, University of California San Diego, La Jolla, CA USA; 5 Cancer Cell Map Initiative (CCMI), University of California San Diego, La Jolla, CA USA; 6 Division of Biomedical Informatics, Department of Medicine, University of California San Diego, La Jolla, CA USA; 7 Department of Quantitative Medicine and Systems Biology, The Translational Genomics Research Institute, Phoenix, AZ USA; 8 Departments of Family Medicine and Public Health and Psychiatry, University of California San Diego, La Jolla, CA USA

**Keywords:** Cancer genomics, Cancer germline, Cancer predisposition, TCGA, Microsatellite instability, Lynch syndrome, Mutational signatures

## Abstract

**Background:**

Cancer susceptibility germline variants generally require somatic alteration of the remaining allele to drive oncogenesis and, in some cases, tumor mutational profiles. Whether combined germline and somatic bi-allelic alterations are universally required for germline variation to influence tumor mutational profile is unclear. Here, we performed an exome-wide analysis of the frequency and functional effect of bi-allelic alterations in The Cancer Genome Atlas (TCGA).

**Methods:**

We integrated germline variant, somatic mutation, somatic methylation, and somatic copy number loss data from 7790 individuals from TCGA to identify germline and somatic bi-allelic alterations in all coding genes. We used linear models to test for association between mono- and bi-allelic alterations and somatic microsatellite instability (MSI) and somatic mutational signatures.

**Results:**

We discovered significant enrichment of bi-allelic alterations in mismatch repair (MMR) genes and identified six bi-allelic carriers with elevated MSI, consistent with Lynch syndrome. In contrast, we find little evidence of an effect of mono-allelic germline variation on MSI. Using MSI burden and bi-allelic alteration status, we reclassify two variants of unknown significance in *MSH6* as potentially pathogenic for Lynch syndrome. Extending our analysis of MSI to a set of 127 DNA damage repair (DDR) genes, we identified a novel association between methylation of *SHPRH* and MSI burden.

**Conclusions:**

We find that bi-allelic alterations are infrequent in TCGA but most frequently occur in *BRCA1/2* and MMR genes. Our results support the idea that bi-allelic alteration is required for germline variation to influence tumor mutational profile. Overall, we demonstrate that integrating germline, somatic, and epigenetic alterations provides new understanding of somatic mutational profiles.

**Electronic supplementary material:**

The online version of this article (10.1186/s13073-018-0579-5) contains supplementary material, which is available to authorized users.

## Background

In rare familial cancer, inherited variation can both increase cancer risk and influence the molecular landscape of a tumor. For example, Lynch syndrome is characterized by an increased cancer risk and increased burden of somatic microsatellite instability (MSI) [1, 2]. The study of this phenomenon has been recently extended to sporadic cancers. For example, carriers of pathogenic mutations in *BRCA1/2* have both increased cancer risk and molecular evidence of homologous recombination deficiency in their tumors [3, 4]. Novel sequencing and analytical methods can be used to reveal a myriad of molecular phenotypes in the tumor, such as mutational signatures, rearrangement signatures, MSI, and infiltrating immune cell content [5–9]. A number of novel associations between these molecular somatic phenotypes and germline variants have recently been discovered. Rare variants in *BRCA1/2* have been associated with mutational signature 3, a novel rearrangement signature, and an overall increased mutational burden [6, 10–12]. Common variants in the *APOBEC3* region have been associated with the corresponding *APOBEC* deficient mutational signature, and a haplotype at the 19p13.3 locus has been associated with somatic mutation of *PTEN* [13, 14]. In addition, interestingly, distinct squamous cell carcinomas (SCCs) arising in the same individual have a more similar somatic copy number profile than SCCs that occur between individuals [15]. Taken together, these results demonstrate that both common and rare germline variation can influence the somatic phenotype of sporadic cancers.

Similar to the two-hit mechanism of inactivation of tumor suppressor genes in familial cancer syndromes described by Nordling and then Knudson decades ago, germline and somatic bi-allelic alteration of *BRCA1/2* is required to induce somatic mutational signature 3, a single germline “hit” is not sufficient [10, 11, 16, 17]. Whether a secondary hit is universally required for germline variation to influence somatic phenotype is currently unclear. Here, we address this question using The Cancer Genome Atlas (TCGA) dataset. TCGA is the most comprehensive resource of germline and somatic variation to enable this analysis, as it contains paired tumor and normal sequence data and a number of other molecular somatic phenotypes for 33 cancer types [18]. In contrast with previous studies of TCGA germline variation that focused on specific cancer types or candidate genes, we performed an exome-wide analysis to identify genes affected by both germline and somatic alterations (referred to as bi-allelic alteration) and study their association with somatic phenotypes [10–13, 19]. Specifically, we conducted an integrated study of all genetic factors that contribute to somatic MSI burden and identified six individuals with characteristics consistent with Lynch syndrome: bi-allelic alteration of a MMR gene, elevated somatic MSI, and an earlier age of cancer diagnosis.

## Methods

### Data acquisition

Approval for access to TCGA case sequence and clinical data were obtained from the database of Genotypes and Phenotypes (project no. 8072, Integrated analysis of germline and somatic perturbation as it relates to tumor phenotypes). Whole exome (WXS) germline variant calls from 8542 individuals were obtained using GATK v3.5 as described previously [20]. The samples prepared using whole genome amplification (WGA) were excluded from the analysis due to previous identification of technical artifacts in both somatic and germline variant calls in WGA samples [20, 21]. Somatic mutation calls obtained using MuTect2 were downloaded from GDC as Mutation Annotation Format (MAF) files [22]. Raw somatic sequence data was downloaded from the Genomic Data Commons (GDC) in Binary Alignment Map (BAM) file format aligned to the hg19 reference genome. Normalized somatic methylation beta values from the Illumina 450 methylation array for the probes most anti-correlated with gene expression were downloaded from Broad Firehose (release stddata__2016_01_28, file extension: min_exp_corr). A total of 7790 samples and 28 cancer types had germline, somatic, and methylation data available.

Segmented SNP6 array data were downloaded from Broad Firehose (release stddata__2016_01_28, file extension: segmented_scna_hg19). Segments with an estimated fold change value ≤ 0.9, which corresponds to a single chromosome loss in 20% of tumor cells, were considered deletions. RNAseq RSEM abundance estimates normalized by gene were downloaded from Broad Firehose (release 2016_07_15, file extension: RSEM_genes_normalized). For 5931 TCGA WXS samples quantitative MSI burden and binary MSI classification calls were obtained from previous work done by Hause et al. [8]. When used as a quantitative phenotype, MSI is expressed as the percentage of microsatellite regions that display somatic instability; when used as a binary classification, MSI is expressed as MSI high (MSI-H) vs. non-MSI. Aggregate allele frequencies and allele frequencies in seven ancestry groups (African, Admixed American, East Asian, Finnish, non-Finnish European, South Asian, and other) were obtained from ExAC v3.01 [23]. Gene-level expression data from normal tissues was downloaded from the GTEx portal (V7, file extension: RNASeQCv1.1.8_gene_tpm) [24].

### Variant annotation and filtering

Raw variant calls were filtered using GATK VQSR TS 99.5 for SNVs and TS 95.0 for indels. Additionally, indels in homopolymer regions, here defined as four or more sequential repeats of the same nucleotide, with a quality by depth (QD) score < 1 were removed.

Putative germline and somatic loss-of-function (LOF) variants were identified using the LOFTEE plugin for VEP and Ensembl release 85 [25]. LOFTEE defines LOF variants as stop-gained, nonsense, frameshift, and splice site disrupting. Default LOFTEE settings were used, and only variants receiving a high confidence LOF prediction were retained. It was further required that LOF variants have an allele frequency < 0.05 in all ancestry groups represented in ExAC. For somatic mutations, LOFTEE output with no additional filters was used. Gene level, CADD score, and ClinVar annotations were obtained using ANNOVAR and ClinVar database v.20170905 [26]. A germline variant was determined to be pathogenic using ClinVar annotations if at least half of the contributing sources rated the variant “Pathogenic” or “Likely Pathogenic.” Li-Fraumeni variant annotations were obtained from the IARC-TP53 database [27–29]. Pfam protein domain annotations used in lollipop plots were obtained from Ensembl BioMart [30, 31].

### Somatic methylation

For each gene, the methylation probe that was most anti-correlated with gene expression was obtained from Broad Firehose and used for all subsequent analyses. Methylation calls were performed for each gene and each cancer type independently. For each gene, the beta value of the chosen methylation probe was converted to a Z-score within each cancer type. Individuals with a Z-score ≥ 3 were considered hyper methylated (*M* = 1), and all others were considered non-methylated (*M* = 0). To determine if methylation calls were associated with reduced somatic gene expression, a linear model of the form log_10_ (*E*_*ij*_)~*C*_*i*_ + *M*_*ij*_ was used, where *E*_*ij*_ denotes expression of gene *j* in tumor *i*, *C*_*i*_ denotes cancer type of sample *i*, and *M*_*ij*_ denotes binary methylation status of gene *j* in sample *i*. Only genes where methylation calls were nominally associated (*p* ≤ 0.05) with decreased gene expression were retained. Using this process, we identified 863,798 methylation events affecting 11,744 genes.

### Loss of heterozygosity

To assess loss of heterozygosity (LOH) for a given heterozygous germline variant, the somatic allele frequency of the germline variant was obtained from the somatic BAM files using samtools mpileup v1.3.1 (SNPs) or varscan v2.3.9 (indels) [32, 33]. Any germline variant that was not observed in the tumor was excluded from further analysis. A one-way Fisher’s exact test comparing reference and alternate read counts was performed to test for allelic imbalance between the normal and tumor sample. Only sites with a nominally significant (*p* ≤ 0.05) increase in the germline allelic fraction were retained. To confirm that the observed allelic imbalance was due to somatic loss of the WT allele and not due to somatic amplification of the damaging allele, we required that the region be deleted in the tumor based on TCGA CNV data (fold change value ≤ 0.9). Loci that had a significant Fisher’s exact test but were not located in a somatic deletion were considered “allelic imbalance” (AI). Using this method, we observed 3418 LOH events in 1672 genes.

### Gene set enrichment analysis

Gene set enrichment analysis was performed using the fgsea R package and the following parameters: minSize = 3, maxSize = 500, nperm = 20,000, and the canonical pathway gene set from MsigDB (c2.cp.v5.0.symbols.gmt) [34, 35]. Genes were ranked according to the fraction of germline LOF variants that acquired a second somatic alteration (number bi-allelic alterations/number germline LOF variants). Genes with fewer than three germline LOF variants in the entire cohort were excluded from this analysis to reduce noise.

### Mutational signature analysis

To identify somatic mutational signatures, counts for each of 96 possible somatic substitutions ± 1 bp context were obtained for all tumor samples. For each sample, mutational signatures were identified using the DeconstructSigs R package, which uses a non-negative least squares regression to estimate the relative contributions of previously identified signatures to the observed somatic mutation matrix [36]. DeconstructSigs was run with default normalization parameters, and relative contributions were estimated for the 30 mutational signatures in COSMIC [37].

To estimate significance of association between germline variants and somatic mutational signature burden, we employed both a pan-cancer Wilcoxon rank sum test and a permutation-based approach to ensure that significance was due to germline variant status and not cancer type. For the permutation approach, the pairing between germline variant status and mutational signature profile was shuffled 10,000×. A Wilcoxon rank sum test was run for each permutation to obtain a null distribution for the test statistic. *P* values were determined for each signature as the fraction of permutations with a Wilcoxon test statistic greater than or equal to the observed data.

### Statistical analyses

Principal component analysis (PCA) was performed on common (allele frequency > 0.01) germline variants using PLINK v1.90b3.29, and the first two principal components obtained from this analysis were used to control for ancestry in all of the regression models we fit to the data [38]. G*Power 3.1 was used to perform a power calculation for the contribution of damaging germline variants to somatic MSI [39]. The following parameters were used: *α* error probability = 0.05, power = 0.80, effect size = 6.83e^−4^, and number of predictors = 20. To assess potential co-occurrence of *SHPRH* methylation with alterations in other genes, individuals were grouped according to presence (+) or absence (−) of *SHPRH* methylation. A one-way Fisher’s exact test was used to test for an abundance of another alteration of interest in *SHPRH* methylation positive individuals vs. *SHPRH* methylation negative individuals. Individuals with > 5000 somatic mutations were excluded from these analyses to exclude potential confounding due to somatic hypermutation.

To test for association between genetic alteration and somatic MSI burden, a linear model of the form log_10_ (*M*_*i*_)~*G*_*ij*_ + *S*_*ij*_ + *Me*_*ij*_ + *X*_*i*_ was used, where *M*_*i*_ denotes somatic MSI burden of sample *i*, *G*_*ij*_, *S*_*ij*_, and *Me*_*ij*_ are binary indicators for germline, somatic, and methylation alteration status of gene *j* in sample *i*, and *X*_*i*_ represents a vector of covariates for sample *i* (cancer type, PC1, PC2). All analyses using somatic MSI data were performed on a maximum of *n* = 4997 individuals. To test for association between germline alteration and age of diagnosis, a linear model of the form *A*_*i*_~*G*_*ij*_ + *X*_*i*_ was used where *A*_*i*_ denotes age of diagnosis for sample *i*, *G*_*ij*_, is a binary indicator for germline alteration status of gene *j* in sample *i*, and *X*_*i*_ represents a vector of covariates for sample *i* (cancer type, PC1, PC2). All analyses using age of diagnosis were performed on a maximum of *n* = 8913 individuals.

## Results

### The MMR pathway is frequently affected by bi-allelic alteration

**Fig. 1:**
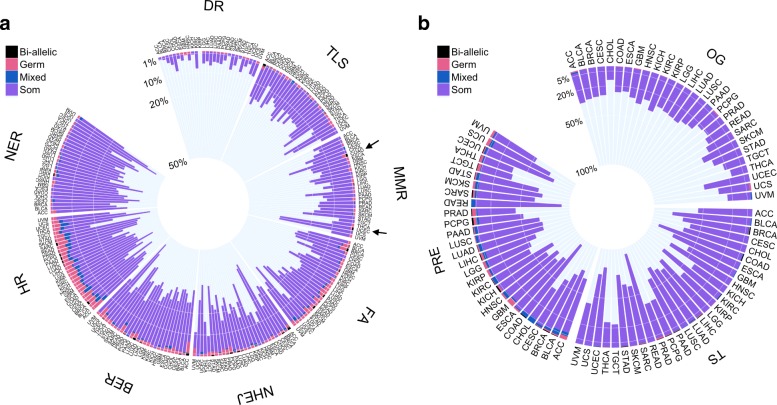
Frequency of germline and somatic alterations in cancer-relevant pathways. **a**–**b** Circos plots displaying the individual-level frequency of alterations for each cancer type in DNA damage repair pathways (**a**) or oncogenes, tumor suppressors, and cancer predisposition genes (**b**). Individuals were grouped into four mutually exclusive categories based on the type of alterations observed in the gene set: Bi-allelic, combined germline and somatic alteration of the same gene; Mixed, germline and somatic alteration of different genes in the set; Germ: germline alterations only; and Som, somatic alterations only (mutation or methylation). The height of each bar represents the fraction of individuals in each alteration category. The black arrows highlight cancer types with bi-allelic mismatch repair alterations. Gene sets are ranked according to size moving clockwise. Pathway abbreviations and sizes: DR direct repair (*N* = 3 genes), TLS translesion synthesis (*N* = 19), MMR mismatch repair (*N* = 27), FA Fanconi anemia (*N* = 34), NHEJ non-homologous end joining (*N* = 37), BER base excision repair (*N* = 43), HR homologous recombination (*N* = 53), NER nucleotide excision repair (*N* = 70), OG oncogenes (*N* = 54), TS tumor suppressors (*N* = 71), and PRE predisposition genes (*N* = 144). There are a total of 382 unique genes, and gene sets are not mutually exclusive

To find events most likely to influence a somatic phenotype, we limited our analysis to alterations predicted to be highly disruptive. We therefore only considered loss-of-function (LOF) germline variants, LOF somatic mutations, epigenetic silencing of genes via DNA hyper-methylation, and somatic loss of heterozygosity (LOH) events that select for a germline LOF allele (see “Methods” and Additional file 1: Figure S1 and S2). In total, we analyzed 7790 individuals with germline variant, somatic mutation, and methylation data available, corresponding to 95,601 germline LOF variants, 225,257 somatic LOF mutations, and 863,798 somatic methylation events (Fig. 1). Using this data, we were able to determine the frequency of three types of germline bi-allelic alterations: (1) germline LOF and somatic LOF (germline:somatic), (2) germline LOF and somatic epigenetic silencing (germline:methylation), and (3) germline LOF with somatic LOH.

Surprisingly, we found a low incidence of bi-allelic alterations, with only 4.0% of all germline LOF variants acquiring a secondary somatic alteration via any mechanism. We observed 198 germline:somatic events (0.02% of all germline LOF), 433 germline:methylation events (0.04%), and 3279 LOH events (3.4%). To determine whether bi-allelic alterations affect specific biological processes, we ranked genes by the frequency of bi-allelic alteration and performed a gene set enrichment analysis (GSEA) using 1330 canonical pathway gene sets [34, 35]. The only association significant beyond a multiple hypothesis correction was an enrichment of germline:somatic alterations in the KEGG mismatch repair (MMR) pathway (*q* = 0.0056) (Additional file 1: Figure S3 and Additional file 2: Table S1). To ensure that the lack of enriched pathways was not due to our strict definition of somatic damaging events, we repeated the analysis including all somatic mutations with a CADD score ≥ 20. Though this increased, the number of germline:somatic alterations (376, 0.039%), no additional significantly enriched pathways were found. Similarly, we repeated the analysis using a less restrictive definition of LOH, referred to as “allelic imbalance” (AI), that accommodates other mechanisms such as copy neutral LOH, subclonal LOH, or intra-tumoral SCNA heterogeneity (see “Methods”). We again observed more AI events (7920, 8.2%), but no additional pathways were significantly enriched.

### Landscape of germline and somatic alteration of DNA damage repair pathways

Having shown that MMR genes frequently harbor bi-allelic alterations, we next investigated the frequency of germline, somatic, and epigenetic alterations in a panel of 210 DNA damage repair (DDR) genes. While germline variation in DDR genes has previously been studied, only a few studies have considered specific DDR pathway information. DDR genes were assigned to eight gene sets using pathway information: direct repair, translesion synthesis, mismatch repair, Fanconi anemia, non-homologous end joining, base excision repair, homologous recombination, and nucleotide excision repair [40]. We also examined three additional cancer-relevant gene sets: oncogenes, tumor suppressors, and cancer predisposition genes (Additional file 3: Table S2) [41, 42]. For each gene set and cancer type, we calculated the fraction of individuals with bi-allelic, germline, somatic, or epigenetic alteration of any gene in the gene set (Fig. 1).

Consistent with previous studies, the fraction of individuals carrying germline LOF was low for both DDR genes and cancer-relevant gene sets (Fig. 1, Additional file 4: Table S3) [12]. Overall, 16% of individuals carried a germline LOF in any of the genes interrogated, with 5% carrying a germline LOF in a known predisposition gene. For each gene set, we tested for overabundance of germline LOF carriers in each cancer type vs. all other cancer types. We discovered associations between breast cancer and germline alteration of the Fanconi anemia and tumor suppressor gene set, which are likely driven by *BRCA1/2* germline variants (Additional file 1: Figure S4a). We expanded our analysis to include known pathogenic missense variants from the ClinVar database and discovered additional significant associations between pheochromocytoma and paraganglioma (PCPG) and both the predisposition and oncogene sets (Additional file 1: Figure S4b and Additional file 5: Table S4) [26]. This association is driven by missense variants in *SDHB* and *RET* that predispose to PCPG and have been previously reported in TCGA [43]. Loss of heterozygosity in these PCPG individuals was frequently observed (77% of *SDHB* germline carriers), consistent with *SDHB* acting via a tumor suppressor mechanism [44]. We conclude that there is no cancer type in TCGA that harbors an excess of damaging germline variants in DDR or cancer-relevant genes, with the exception of the well-described predisposition syndrome genes *BRCA1/2*, *SDHB*, and *RET*.

### A subset of individuals in TCGA exhibits characteristics of Lynch syndrome

**Fig. 2:**
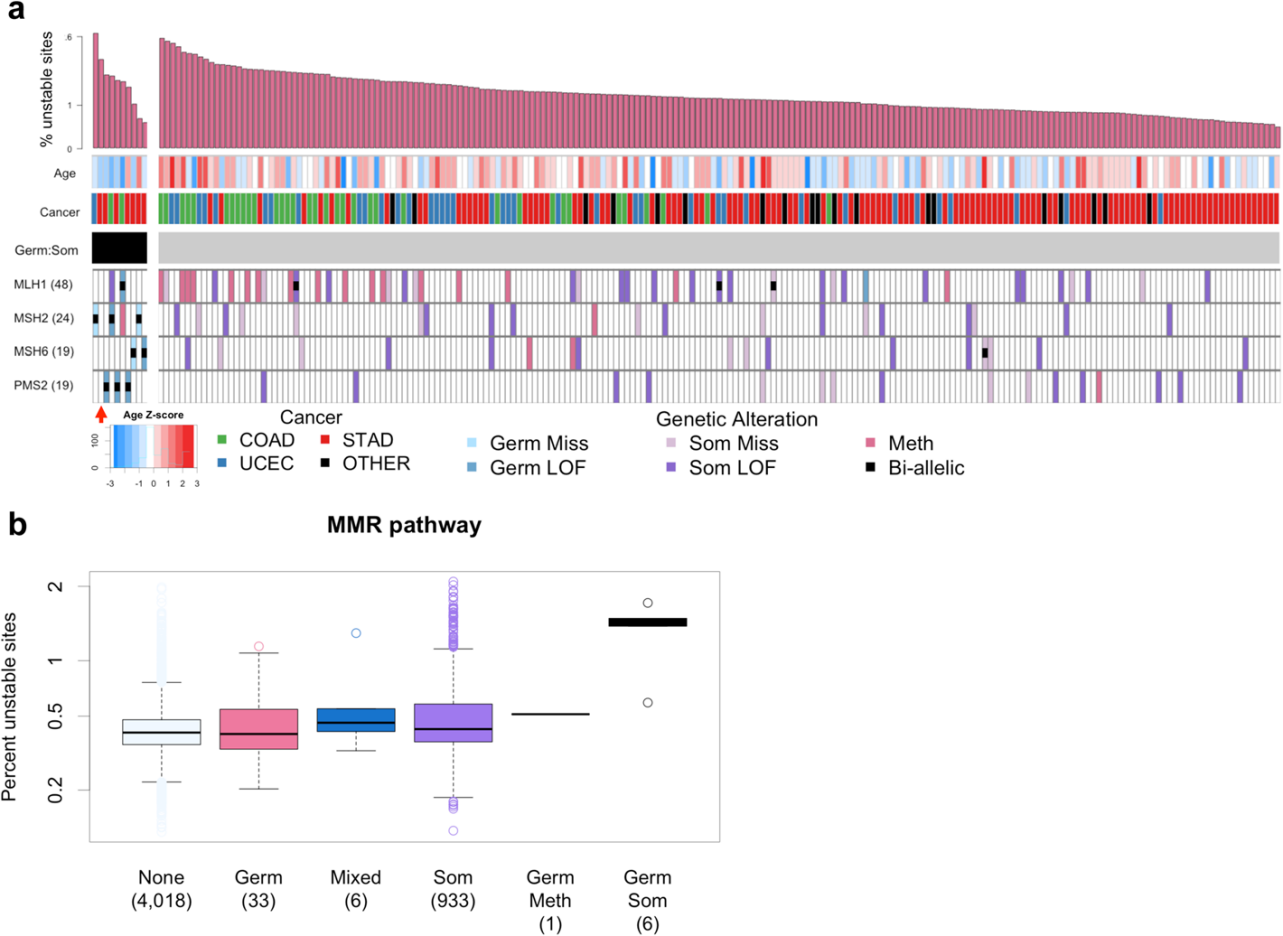
Genetic and clinical characteristics of MSI-H individuals. **a** CoMut plot displaying germline, somatic, and epigenetic events in L-MMR genes (bottom 4 rows—number of affected individuals in parentheses) for 217 MSI-H individuals (columns). The top histogram represents MSI burden expressed as the fraction of possible microsatellite sites that are unstable. Age of diagnosis was converted to a Z-score using the mean and standard deviation age for each cancer type. Cancer types with fewer than 5 MSI-H individuals are labeled “Other” and include bladder, head and neck, kidney, glioma, lung, liver, prostate, stomach, and rectal cancer. The type of genetic alteration is indicated by color, and bi-allelic events are indicated by a black box. Individuals with bi-allelic (germline:somatic) MMR mutations are grouped to the left. The red arrow highlights an individual with bi-allelic alteration in *MSH5* (not an L-MMR gene). **b** Somatic MSI burden in 4997 TCGA individuals grouped by type of MMR pathway alteration. Categories are the same as those described in Fig. 1: Bi-allelic, combined germline and somatic alteration of the same gene; Mixed, germline and somatic alteration of different genes in the set; Germ, germline alterations only; and Som, somatic alterations only (mutation or methylation). Individuals with bi-allelic alteration occurring via germline:somatic and germline:methylation mechanisms are displayed separately. The number of individuals in each category is indicated in parentheses

We found that the MMR pathway was significantly enriched for germline:somatic alterations. This association was driven by six individuals who carry a germline:somatic alteration of a MMR gene. In five individuals, the gene affected was a known Lynch syndrome gene (*MLH1*, *MSH2*, *MSH6*, and *PMS2*), which we will refer to as L-MMR genes [2]. The remaining individual carried a germline:somatic alteration of *MSH5* (Fig. 2a, red arrow). While *MSH5* is not known to be a Lynch syndrome gene, we included this individual in further analyses of MMR germline:somatic alteration carriers. Four of the germline:somatic alteration carriers have uterine cancer (UCEC) and two have colon cancer (COAD), cancer type characteristic of Lynch syndrome (Fig. 1b, arrows) [45]. This prompted us to investigate the molecular and clinical phenotype of germline:somatic alteration carriers to determine if they are consistent with Lynch syndrome characteristics. While germline:somatic alteration of MMR genes in TCGA has been previously described, detailed somatic phenotyping of these individuals has not been performed [9]. Using previously published MSI data, we investigated the fraction of microsatellite loci that exhibit instability in the tumor (somatic MSI burden) of individuals carrying alterations in MMR genes [8]. Figure 2a shows germline, somatic, and epigenetic alteration status of L-MMR genes for all individuals classified as MSI high (MSI-H) by Hause et al., with bi-allelic mutation carriers grouped to the left. Interestingly, only 76% of MSI-H individuals have an alteration (germline LOF, somatic LOF, or hyper-methylation) of an MMR gene, indicating that some of the variation in somatic MSI is not explained by the genetic alterations investigated.

Using a linear model controlling for cancer type, we found that the 6 individuals with germline:somatic MMR alterations were diagnosed on average 14 years earlier (*p* = 0.0041) and have 2.8 fold higher somatic MSI (*p* = 3.95e^−15^) than individuals with any other type of MMR pathway alteration (Fig. 2b, Additional file 1: Tables S5, S6). Of the five individuals with germline:somatic alteration of a L-MMR gene, four carried a germline LOF variant that is known to be pathogenic for Lynch syndrome, and one carried a LOF variant *MSH6* (p.I855fs) not present in ClinVar (Additional file 1: Table S7). This frameshift *MSH6* VUS is five base pairs upstream of a known pathogenic frameshift variant. This suggests that disruption of the reading frame in this gene region is pathogenic and the novel MSH6 variant likely also predisposes to Lynch syndrome (Additional file 1: Table S8). While a diagnosis of Lynch syndrome requires clinical family history data not available in TCGA, the carriers were diagnosed at an earlier age and exhibit increased somatic MSI characteristic of Lynch syndrome. We note that this result would have gone unnoticed in an analysis of somatic MSI using interaction terms to model bi-allelic alteration at the single gene level, highlighting the value of grouping genes by biological pathway (Additional file 1: Table S9). Interestingly, we observed the identical nonsense mutation in *PMS2* (p.R628X) in two individuals, once as an inherited variant and once as an acquired somatic mutation (Additional file 1: Figure S5). This overlap between clinically relevant germline variants and somatic mutations suggests that, in some instances, the origin of a mutation is less important than its functional effect.

### Using the MSI-H phenotype to identify potentially pathogenic variants

Given the large effect of germline:somatic LOF mutations on somatic MSI, we next asked whether germline:somatic missense mutations produced a similar phenotype. We expanded our analysis to include missense variants known to be pathogenic for Lynch syndrome from ClinVar. We identified one individual with bi-allelic alteration of *MSH2* involving a pathogenic missense germline variant (p.S554 N) and a somatic LOF mutation (Additional file 1: Table S7). Including missense somatic mutations with a CADD score ≥ 20 led to the identification of one individual with bi-allelic alteration of *PMS2* involving a germline LOF variant (p.R563X) and a secondary somatic missense mutation (Additional file 1: Table S8).

**Fig. 3:**
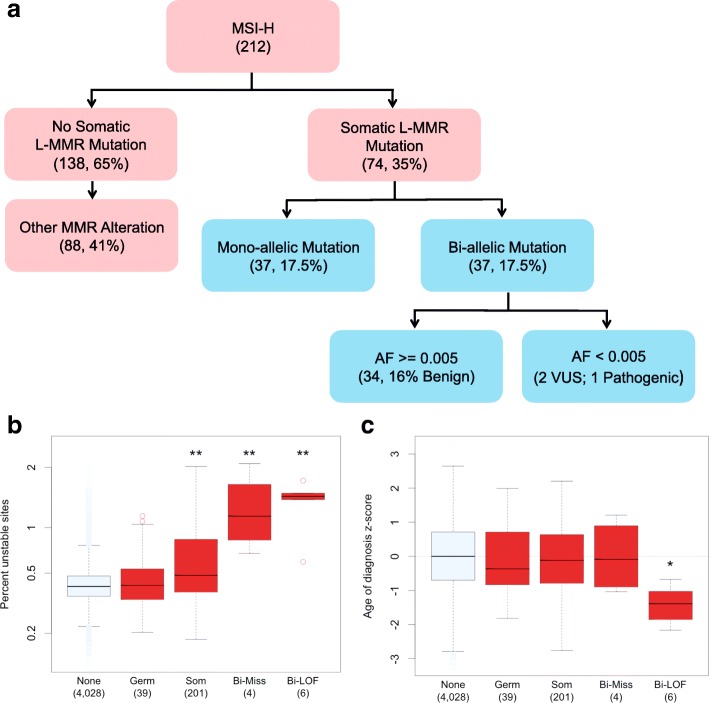
Identification and characterization of potential pathogenic Lynch syndrome variants. **a** Analysis workflow: 212 individuals with MSI-H classification were dichotomized based on the presence of germline:somatic mutation of a L-MMR gene. Individuals carrying germline:somatic mutations were further subdivided by allele frequency of the candidate germline variant in ExAC. Pink boxes indicate the use of somatic data, and blue boxes integrate somatic and germline data. Numbers in parentheses refer to number of individuals that fulfill the box criteria. Individuals that carry bi-allelic alterations are labeled according to ClinVar significance of the germline variant. VUS variant of unknown significance. **b**, **c** Somatic MSI burden (**b**) and age of diagnosis (**c**) of individuals who carry germline:somatic mutations in a MMR gene. Individuals were grouped by MMR gene mutation type: None, no alteration; Germ, germline LOF variants only, Som, somatic LOF mutations only; Bi-Miss, bi-allelic alteration including a missense mutation; and Bi-LOF, bi-allelic alteration via dual LOF mutations. Age was converted to a Z-score using the mean and standard deviation age of diagnosis for each cancer type. **p < 0.001, *p < 0.01; *p* values were determined using a linear model to predict somatic MSI burden while accounting for cancer type

We observed a number of missense germline variants in L-MMR genes not present in ClinVar, which we consider variants of unknown significance (VUS). We reasoned that the phenotype of elevated somatic MSI and germline:somatic L-MMR mutation could be used to identify germline VUS likely to be pathogenic for Lynch syndrome. Using 212 individuals classified as MSI-H, we identified 74 individuals with a damaging somatic mutation in a L-MMR gene (Fig. 3a) [8]. Of the individuals with L-MMR somatic mutations, 37 have a germline missense variant in the somatically mutated gene. To identify variants most likely to be damaging, we retained only those with a minor allele frequency < 0.005 in all ancestry groups represented in ExAC. Three individuals met the criteria of having an MSI-H phenotype and a bi-allelic L-MMR mutation involving a likely damaging missense germline variant. One was the previously identified *MSH2* p.S554N variant carrier, the others carried two VUS: *MSH2* (p.P616R) and *MSH6* (p.F432C) (Additional file 1: Table S8).

Closer investigation of the *MSH6* p.F432C variant showed that other amino acid substitutions at the same residue were classified as pathogenic in ClinVar (Additional file 1: Table S8). Should these VUS be pathogenic, we would expect the carriers to have an earlier age of cancer diagnosis. The individual carrying the *MSH6* p.F432C variant was diagnosed earlier than average (*Z* = − 1.03) while the individual carrying the *MSH2* p.P616R variant was diagnosed later (*Z* = 1.20). Age of diagnosis cannot be used alone to classify a variant; however, this evidence suggests that *MSH2* p.P616R may not be pathogenic. While validation is required to confirm pathogenicity of this variant as well as the previously mentioned *MSH6* p.I855fs, we offer evidence that these variants may predispose to Lynch syndrome, as well as show evidence suggesting that *MSH2* p.P616R may be benign.

### Missense bi-allelic alterations exhibit an attenuated phenotype

Taken together, we have identified ten individuals with germline:somatic MMR alterations, six of which carry a germline variant that is known to be pathogenic for Lynch syndrome (Table 1). With this in mind, we asked whether individuals with germline:somatic LOF mutations have a more severe phenotype than those with combined LOF and missense mutations. Bi-allelic alteration carriers were divided into two groups: those with germline and somatic LOF mutations (Bi-LOF, *n* = 6) and those with missense germline variants or missense somatic mutations (Bi-Miss, *n* = 4). We found that both Bi-LOF (*p* = 2.78e^−15^) and Bi-Miss (*p* = 1.01e^−10^) groups have significantly elevated MSI (Fig. 3b and Additional file 1: Table S10). Bi-Miss and Bi-LOF have a median 1.50 and 2.35 fold higher somatic MSI compared to individuals with somatic MMR alteration alone, demonstrating a synergistic effect between germline variants and somatic mutations. Similarly, both Bi-LOF and Bi-Miss groups had significantly higher contribution of mutational signature 6, a signature associated with mismatch repair defects (Additional file 1: Figure S6) [7]. In contrast, only Bi-LOF individuals were diagnosed at an earlier age (Fig. 3c and Additional file 1: Table S11). These results show that any damaging bi-allelic MMR alterations are sufficient to induce high levels of somatic MSI, but only bi-allelic alterations via dual LOF mutation are associated with an earlier age of diagnosis.

**Table 1:** Number of individuals affected by three types of germline:somatic alterations in MMR genes

Gene	Germline LOF somatic LOF	Germline LOF somatic MISS	Germline MISS somatic LOF
*MLH1*	1*		
*MSH2*	1*		1,1*
*MSH6*	1		1
*PMS2*	2*	1*	
*MSH5*	1		

*Individual carries a ClinVar pathogenic germline variant

### Mono-allelic damaging germline alteration has minimal effect on somatic MSI burden

Having shown that combined germline LOF and missense somatic mutations are sufficient to cause elevated MSI, we hypothesized that damaging germline variation in the absence of somatic mutation could also increase somatic MSI. To maximize power, we expanded our analysis to include all MMR genes as well as two different categories of damaging germline variation: known (ClinVar) and predicted (CADD ≥ 30) pathogenic (Additional file 5: Table S4). Individuals with any somatic alterations in MMR genes were excluded from this analysis to get an accurate estimate of the effect of damaging germline variation alone. There were no significant association between damaging germline variation in the MMR pathway and somatic MSI burden (Additional file 1: Figure S7 and Table S12). Known variants showed the strongest effect (0.02 fold increase in MSI burden), and this was largely driven by *MLH3* p.V741F, a variant with conflicting reports of pathogenicity that is carried by 195 individuals. From this, we conclude that the effect of damaging germline variation without concomitant somatic mutation on somatic MSI is small.

### Methylation of *SHPRH* associated with somatic MSI burden

**Fig. 4:**
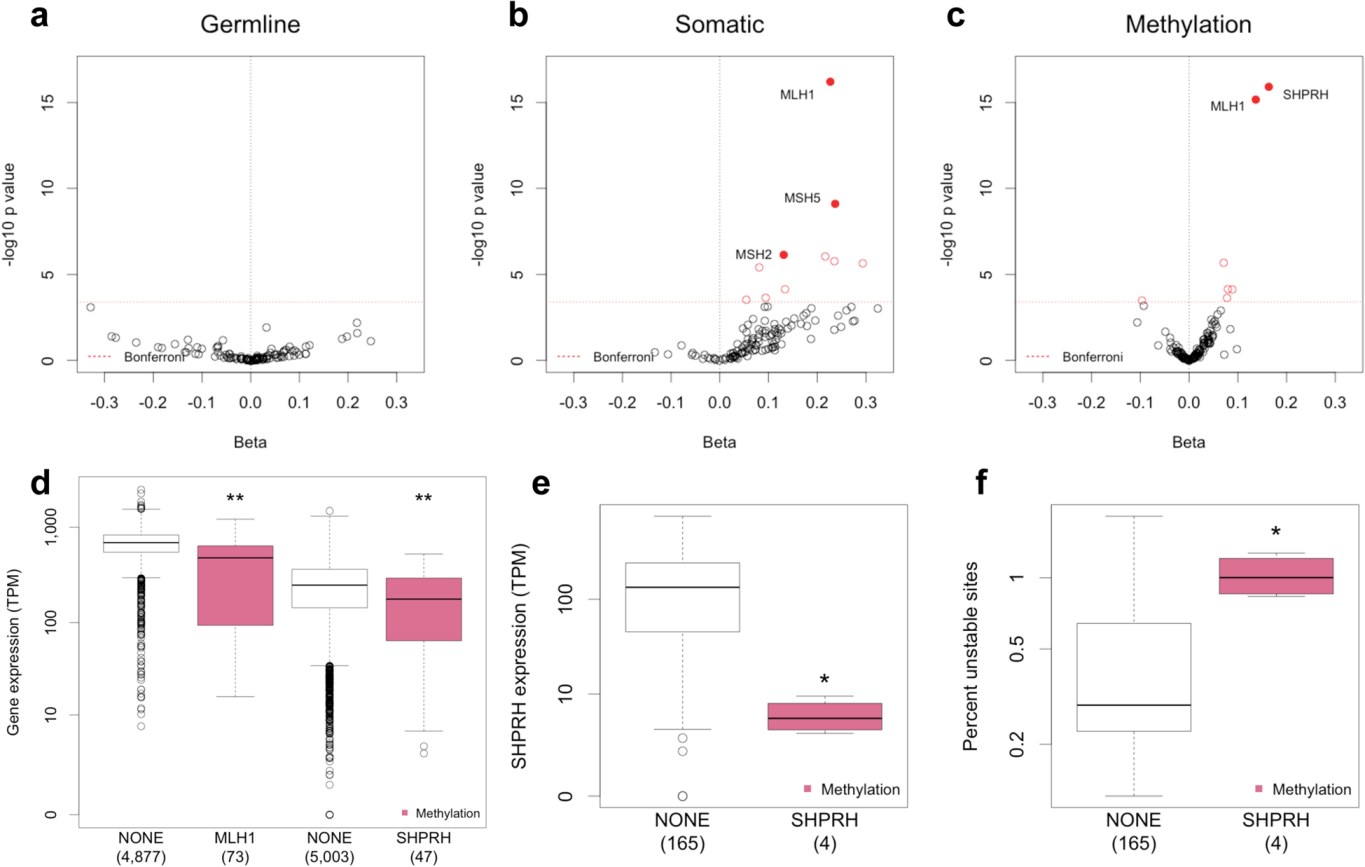
Germline, somatic, and epigenetic alterations that influence somatic MSI burden. **a**–**c** Volcano plots of gene-level association testing between germline LOF (**a**) somatic LOF (**b**) and somatic methylation (**c**) and somatic MSI burden. A total of 127 DDR genes were tested in 4987 individuals. Red dotted line represents Bonferroni significance cutoff. **d** Somatic expression of *MLH1* and *SHPRH* in individuals with somatic methylation. **p < 0.001 as determined using a linear model to predict gene expression while accounting for cancer type. **e**, **f** Somatic *SHPRH* expression is significantly reduced (**e** Wilcox *p* = 0.0018), and somatic MSI is significantly increased (**f**, Wilcox *p* = 0.0067) in uterine tumors with *SHPRH* methylation. TPM transcripts per million. The number of individuals in each category is indicated in parentheses

We observe that 24% of MSI-H individuals have no alteration (germline LOF, somatic LOF, or hyper-methylation) of an MMR gene, suggesting that there is variation in somatic MSI burden due to factors outside of known MMR genes (Fig. 3b) [46]. To investigate this further, we extended the search to all DDR genes. We separately assessed the contribution of germline LOF, somatic LOF, and somatic methylation to somatic MSI burden using a gene level linear model. Somatic LOF frameshift mutations that overlap with microsatellite loci were removed from this analysis, as we were unable to determine the direction of causality between these mutations and overall MSI burden (Additional file 1: Figure S8 and Table S13). Additionally, the MMR bi-allelic alteration carriers were excluded from this analysis to obtain an accurate assessment of mono-allelic germline variation. The results of this analysis are summarized in Fig. 4. Consistent with the lack of association between damaging MMR germline variants and somatic MSI, we found no significant association at the single gene level between germline LOF and somatic MSI (Fig. 4a).

We found that somatic mutation of *MLH1* and *MSH2* and somatic methylation of *MLH1* were associated with increased MSI burden, confirming what has been previously reported (Fig. 4b, c) [46]. In addition, we discovered a novel association between methylation of *SHPRH* and elevated somatic MSI (*p* = 1.19e^−16^) (Fig. 4c). *SHPRH* is a E3 ubiquitin-protein ligase and a member of the translesion synthesis pathway, a pathway that enables DNA replication to traverse regions of DNA damage via specialized polymerases [47]. Methylation of *SHPRH* was associated with a 16% decrease in gene expression in a pan-cancer analysis (Fig. 4d). We observed that methylation of *SHPRH* has the strongest effect both on *SHPRH* expression and somatic MSI burden in uterine cancer (Fig. 4e, f and Additional file 1: Figure S9). Interestingly, *SHPRH* expression is highest in normal ovarian and uterine tissues among 23 tissues examined, suggesting a specific function for *SHPRH* in these organs (Additional file 1: Figure S10) [24]. Methylation of *MLH1* and *SHPRH* are both associated with mutational signature 6, with a stronger association in uterine cancer (Additional file 1: Figure S11).

To confirm that *SHPRH* methylation is the likely causal factor influencing somatic MSI, we performed a co-occurrence analysis to find other somatic events correlated with *SHPRH* methylation (Additional file 1: Figure S12). There were a large number of somatic events significantly correlated with *SHPRH* methylation, including somatic MMR mutations; however, we found that *SHPRH* methylation remains a significant determinant of somatic MSI even after accounting for other somatic MMR alterations (Additional file 1: Table S14). Furthermore, we found a significant, albeit weaker, association between somatic expression of *SHPRH* and MSI burden, indicating that *SHPRH* methylation likely affects MSI burden via silencing of *SHPRH* (Additional file 1: Table S15).

### Mono-allelic germline alterations are not associated with somatic mutational signatures

We demonstrate that bi-allelic alteration is necessary for germline variants to influence somatic MSI. Next, we investigated whether this requirement for bi-allelic alteration applied to other somatic phenotypes, such as mutational signatures. We hypothesized that mono- or bi-allelic alterations in other DDR pathways may also be associated with known mutational signatures, as has been demonstrated between bi-allelic alteration of *BRCA1/2* and mutational signature 3 [10]. We first attempted to replicate the *BRCA1/2* association, but surprisingly found high levels of mutational signature 3 in individuals carrying mono-allelic damaging germline *BRCA1/2* variation. However, when we considered AI events to be bi-allelic alterations, we no longer found a significant association between mono-allelic *BRCA1/2* alterations and somatic mutational signature 3 (Additional file 1: Figure S13 and Additional file 6: Table S16). In contrast to individuals with *BRCA1/2* LOH, we suspect that individuals with AI have subclonal *BRCA1/2* loss, which would explain the lower levels of signature 3 observed. Thus, we demonstrate that variability in LOH calling method can lead to conflicting results.

We next tested for association between 30 somatic mutational signatures from COSMIC and germline bi-allelic alteration in six DDR pathways with more than five individuals carrying bi-allelic alteration (FA, MMR, HR, BER, NHEJ, and TLS) (Additional file 1: Figure S14a) [37]. The only significant association uncovered (FDR < 15%) was between Fanconi anemia and signature 3, which was driven by the known association between *BRCA1/2* alterations and signature 3. We found that when we include all bi-allelic alterations in MMR genes, there was no significant association with signature 6. This was due to the inclusion of germline:methylation events. Limiting our analyses to germline:somatic events led to an association that was statistically significant after multiple hypothesis correction (Additional file 1: Figure S6). This suggests that the mechanism of secondary somatic alteration modulates the effect of germline variation on somatic phenotype. We repeated this analysis expanding to include individuals with mono-allelic germline alteration in DDR pathways and found no significant associations (Additional file 1: Figure S14b). While this analysis is limited due to the small number of individuals carrying pathogenic germline variants, our results are consistent with the previously established idea that bi-allelic alteration is required for the germline to alter somatic mutational phenotypes.

### Cancer predisposition syndromes in TCGA

**Fig. 5:**
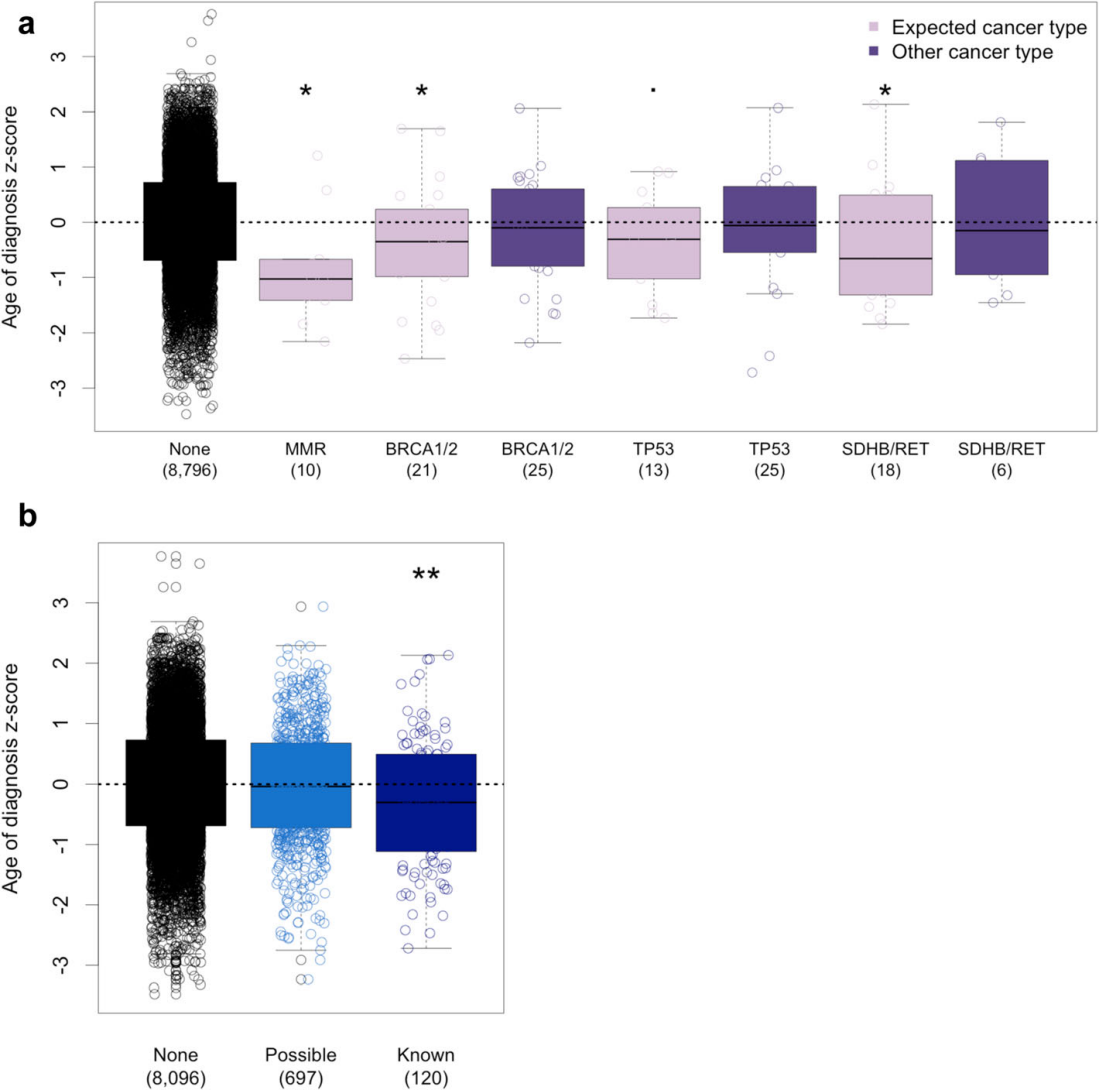
Cancer predisposition syndromes in TCGA. **a** Age of diagnosis for MMR germline:somatic alteration carriers and individuals carrying ClinVar pathogenic or LOF germline variation in *BRCA1*, *BRCA2*, *TP53*, *SDHB*, and *RET*. Age was converted to a Z-score using the mean and standard deviation age of diagnosis for each cancer type. The expected cancer types for each gene set are MMR, colon, uterine, and stomach; *BRCA1/2*, breast cancer; *TP53*, adrenal cortical carcinoma, glioma, glioblastoma, breast cancer, and sarcoma; and *SDHB/RET*, pheochromocytoma, and paraganglioma. All MMR germline:somatic alteration carriers have the expected cancer type. The number of individuals in each category is displayed in parentheses. **b** Age of diagnosis for individuals carrying ClinVar pathogenic or LOF germline variation in genes described in **a** (“known”) compared to a set of 75 other cancer predisposing genes (“possible”). **p < 0.001, *p < 0.05, *p* < 0.1. *p* values were determined using a linear model to predict age of onset while accounting for cancer type

While TCGA is generally thought to represent sporadic adult-onset cancers, our work as well as that of others has shown evidence suggesting that some individuals in TCGA have hereditary cancer predisposition syndromes. Known pathogenic variation in *SDHB/RET*, *BRCA1/2*, and MMR genes is thought to be responsible for a subset of pheochromocytoma and paraganglioma, breast, ovarian, colon, and uterine cancers in TCGA [9, 10, 43, 48]. Another relatively common cancer syndrome that predisposes to cancer types found in TCGA is Li-Fraumeni syndrome (LFS), which arises due to inherited variation in TP53 [1]. Using the IARC-TP53 variant database, we identified 38 individuals carrying a potential LFS variant (Additional file 5: Table S4). Interestingly, aside from bi-allelic MMR alteration, we observed that pathogenic germline variation in cancer predisposition genes was not associated with an earlier age of diagnosis in 8913 individuals with both germline and age of diagnosis data available. To explore this further, we divided individuals into two groups: those who developed the cancer type expected given the predisposition gene altered and those with another cancer type. Using this approach, we found significant associations between germline alteration status and age of diagnosis for the expected cancer type (Fig. 5a and Additional file 1: Table S17). This suggests that predisposition syndromes can lead to an earlier age of onset in a specific spectrum of cancers, but have no significant effect on other cancer types.

To determine if damaging germline variation in other predisposition genes was associated with earlier age of diagnosis, we examined 75 cancer predisposition genes not included in the previous analysis. We found no significant association between germline alteration status and age of diagnosis in any of these additional genes (Additional file 1: Figure S15 and Table S18). To increase power, we examined these additional genes in aggregate as a gene set (“possible”) and compared this gene set to the genes we examined previously (“known,” *BRCA1*, *BRCA2*, *MLH1*, *MSH2*, *MSH5*, *MSH6*, *PMS2*, *SDHB*, *RET*, and *TP53*). The known gene set was associated with an earlier age of diagnosis, but the possible gene set was not (Fig. 5b). It is possible that using biological knowledge to group genes or cancer types in a meaningful way could increase power and find new associations. However, we believe much of the variation in age of diagnosis due to germline variation lies in genes associated with prevalent cancer predisposition syndromes.

## Discussion

We present an analysis of cancer exomes that integrates germline variation, somatic mutation, somatic LOH, and somatic methylation. To our knowledge, our study is the first exome-wide analysis of the prevalence of bi-allelic alterations across the full spectrum of cancer types represented in TCGA and one of the first to integrate somatic methylation data for a large number of genes. Of all gene sets and bi-allelic alteration mechanism examined, we only discovered a significant enrichment of combined germline and somatic LOF mutations in the MMR pathway. Bi-allelic alteration of the MMR pathway has been previously reported; however, the individuals harboring these alterations were not studied in detail [9]. While a diagnosis of Lynch syndrome cannot be made without a family history, we identified ten individuals with bi-allelic alteration in an MMR gene, elevated somatic MSI burden, and, in individuals with bi-allelic LOF mutations, earlier age of cancer diagnosis.

The genes harboring bi-allelic alterations by our analyses are predominantly those that are less frequently mutated in Lynch syndrome: *MSH6* and *PMS2*. Similarly, only 20% of the proposed Lynch individuals have colon cancer, the classic Lynch presentation. Thus, it is possible that what we observe is not bona fide Lynch syndrome, but an attenuated form of the disease [45, 49]. The median age of cancer onset in TCGA is 60; thus, the individuals in TCGA carrying cancer predisposing variants may have genetic modifier mechanisms that delay cancer onset and severity. Interestingly, proposed mechanisms of genetic compensation delaying cancer onset have been described previously both for Lynch syndrome and Li-Fraumeni syndrome [50, 51]. We observed six individuals carrying a potentially pathogenic germline variant in a L-MMR gene (two ClinVar pathogenic, four LOF) who did not acquire a second somatic mutation and do not have elevated somatic MSI burden. This is not unexpected as the penetrance of Lynch syndrome variants is often incomplete [2]. We observed that any damaging germline:somatic alteration is sufficient to induce elevated somatic MSI, but only individuals with Bi-LOF mutation have an earlier age of diagnosis. This observation is consistent with the previously proposed idea that bi-allelic MMR mutation is likely not the tumor-initiating event but instead acts to accelerate tumor growth (Fig. 3b, c) [2]. Given our observations, we propose that the less damaging Bi-Miss mutations could lead to slower tumor growth than Bi-LOF mutations.

Recently, Polak et al. demonstrated that somatic mutational signature 3 and *BRCA1/2* LOH bi-allelic inactivation could be used to reclassify *BRCA1/2* germline variants that were previously considered VUS [10]. Here, we provide another example of how somatic phenotype data can be used to reclassify germline VUS. We identify two novel potentially damaging Lynch syndrome variants in *MSH6*. Of note, the ClinVar pathogenic Lynch predisposing *MSH2* variant was not present in the ANNOVAR ClinVar database despite being reported in ClinVar, highlighting the importance of manual curation of potentially pathogenic variants. Further experimental validation of these variants is required. Germline MMR variants can be used to guide therapy and monitoring for patients at risk. For example, the risk of colorectal cancer can be reduced in individuals carrying pathogenic germline MMR variants using a daily aspirin regimen [42, 52]. Distinguishing between sporadic cancer and cancer driven by inherited variation is important both for treatment of the individual as well as for informing relatives who may carry the same inherited predisposition. The novel variants we discovered could increase the knowledge base of variants that predispose to cancer.

A large portion of population-level variation in MSI is not easily explained by germline, somatic, or epigenetic alteration in DDR genes. This could be due to our modeling approach, our strict criteria for defining damaging events, copy number events we did not analyze, measurement error in the evaluation of the MSI phenotype, or the limited focus on DDR genes. Despite these constraints, we successfully identified a novel association between methylation of *SHPRH* and somatic MSI burden, with a particularly strong effect in uterine cancer where *SHPRH* methylated individuals exhibit a 2.4 fold increase in somatic MSI burden. This finding is particularly interesting as outside of *MLH1*, and there is little evidence of other epigenetic alterations associated with somatic MSI burden [53, 54]. Knockdown of *SHPRH* in yeast has previously been shown to increase DNA breaks and genomic instability [55]. To our knowledg*e*, *SHPRH* has not been directly associated with MSI and therefore should motivate further biological validation of this result.

The lack of significant GSEA hits from the exome-wide bi-allelic alteration analysis suggests that there are few novel genes to be found using TCGA that fit the two-hit inactivation model proposed by Nording and Knudson [16, 17]. However, we recognize that our methodology for calling LOH is simplistic and that more sophisticated methods can better identify complex LOH events, for instance copy neutral LOH. We illustrate how differences in LOH calling methodology for germline *BRCA1/2* variants can lead to conflicting conclusions about the frequency of bi-allelic alteration (Additional file 1: Figure S13). Therefore, it is possible that more sophisticated methods may discover novel genes frequently affected by bi-allelic alteration. Outside of bi-allelic alteration, we find that mono-allelic damaging germline variation has little effect on somatic MSI burden. This is not entirely surprising, as there is conflicting evidence on the effect of MMR haploinsufficiency on mutation rates [45, 56]. Using the effect size of known pathogenic MMR variants, we performed a power calculation and estimated that 11,482 individuals (6485 more than our analysis) would be required to detect the association between mono-allelic damaging germline MMR variants and somatic MSI (see “Methods”). We further found no significant association between mono-allelic damaging germline variants and somatic mutational signatures. Our analysis suggests that the contribution of mono-allelic germline variation to somatic mutational phenotypes is likely to be small.

In addition to individuals with potential Lynch syndrome, we identified individuals who carry germline variants that reportedly predispose to Li-Fraumeni spectrum cancers as well as pheochromocytoma and paraganglioma. While the number of individuals who carry these variants is small, in some cases, their phenotype is extreme enough to confound analyses, as we saw with somatic MSI (Additional file 1: Figure S8b and Table S13). It is important that studies using TCGA as a sporadic cancer control remove potential confounding cases [57]. These individuals may have escaped previous notice due to the fact that many did not develop the cancer type expected based on their germline predisposition. This confirms the variable penetrance of some variants associated with predisposition syndromes: a variant can predispose to one cancer type but have no significant effect on the course of disease of another cancer type [42]. Some individuals with an inherited predisposition variant will not acquire the cancer type they are predisposed toward, but “bad luck” or environmental exposures will lead them to develop a sporadic cancer [58, 59].

## Conclusions

The goal of this study was to assess the ability of germline mono-allelic and germline and somatic combined bi-allelic alterations to alter somatic molecular phenotypes. We observed that combined germline and somatic alteration of MMR genes had a synergistic effect on somatic MSI burden, but germline alteration alone showed no effect. We later showed that germline variation in known cancer predisposition genes only led to an earlier age of diagnosis only in a subset of cancer types. From these observations, we conclude that germline variation has the ability to influence both somatic phenotypes and cancer development, but often, this ability is dependent on other somatic alterations or tissue type-specific processes. Our work highlights the importance of integrating germline and somatic data to identify bi-allelic alterations when testing for associations between germline variants and somatic phenotypes.

In this study, we intended to characterize sporadic adult-onset cancers, but in the course of our analyses, we identified individuals that likely have rare cancer predisposition syndromes. Our results and observations shed important light on the issue of incidental findings, not only in the TCGA, but also in any dataset with paired germline variant and phenotype data. We have taken care to be sensitive in our reporting of the data for patient privacy and followed precedents set by others using the TCGA germline data. We believe it will be important moving forward to have a set standard for reporting germline variation, especially given the recent surge of interest in germline variation in cancer.

## Additional files

Additional file 1: **Figure S1.** Calling somatic methylation status. **Figure S2.** Example LOH events. **Figure S3.** Genes frequently affected by germline:somatic alteration. **Figure S4.** Association between germline LOF burden and cancer type. **Figure S5.** Both germline and somatic LOF mutations can alter the same position. **Figure S6.** Mutational signature analysis of germline:somatic MMR alteration carriers. **Figure S7.** Mon-allelic germline variation in MMR pathway not associated with somatic MSI. **Figure S8.** Association testing between germline, somatic, and epigenetic alteration and somatic MSI burden. **Figure S9.** *SHPRH* methylation in uterine cancer. **Figure S10.** *SHPRH* expression in normal tissues. **Figure S11.** Mutational signature analysis of *MLH1* and *SHPRH* methylated samples. **Figure S12.** Co-occurrence testing for *SHPRH* methylation. **Figure S13.** Mutational signature analysis of *BRCA1/2* germline variant carriers. **Figure S14.** Mutational signature analysis of mono- and bi-allelic alteration of DDR pathways. **Figure S15.** Association between damaging germline variants and age of diagnosis. **Table S6.** Association between age of diagnosis and MMR pathway alteration. **Table S7.** ClinVar annotations for germline variants pathogenic for Lynch syndrome. **Table S8.** ClinVar annotations for germline variants of unknown significance. **Table S9.** Modeling a gene-level germline:somatic interaction for L-MMR genes. **Table S10.** Association between type of germline:somatic mutation and somatic MSI burden. **Table S11.** Association between germline:somatic mutation types and age of diagnosis. **Table S12.** Association between mono-allelic germline MMR variants and somatic MSI burden. **Table S13.** MSI linear model results using unfiltered somatic mutations and with germline:somatic MMR alteration carriers included. **Table S14.** Modeling somatic MSI burden using MMR perturbations highly correlated with *SHPRH* methylation. **Table S15.** Modeling somatic MSI burden using *SHPRH* expression. **Table S17.** Association between MMR, *BRCA1/2*, *SDHB/RET*, and *TP53* germline variant carrier status and age of diagnosis. **Table S18.** Association between predisposition gene germline variant carrier status and age of diagnosis. (PDF 2401 kb)

Additional file 2: **Table S1.** Gene set enrichment analysis on gene-level frequency of bi-allelic alteration. (XLS 90 kb)

Additional file 3: **Table S2.** List of genes and pathways used. (XLS 27 kb)

Additional file 4: **Table S3.** Gene-level frequency of mono- and bi-allelic germline alterations. (XLS 44 kb)

Additional file 5: **Table S4.** List of ClinVar, IARC-TP53, and CADD damaging germline variants used. (XLS 836 kb)

Additional file 6: **Table S16.** *BRCA1/2* LOH calls using different LOH calling approaches. (XLS 51 kb)

## Abbreviations

AI: Allelic imbalance
BER: Base excision repair
COAD: Colon cancer
DDR: DNA damage repair
DR: Direct repair
FA: Fanconi anemia
GDC: Genomic Data Commons
GSEA: Gene set enrichment analysis
HR: Homologous recombination
LFS: Li-Fraumeni syndrome
LOF: Loss-of-function
LOH: Loss of heterozygosity
MAF: Mutation Annotation Format
MMR: Mismatch repair
MSI: Microsatellite instability
MSI-H: MSI high
NER: Nucleotide excision repair
NHEJ: Non-homologous end joining
OG: Oncogenes
PCA: Principal component analysis
PCPG: Pheochromocytoma and paraganglioma
PRE: Predisposition genes
QD: Quality by depth
SCC: Squamous cell carcinoma
TCGA: The Cancer Genome Atlas
TLS: Translesion synthesis
TS: Tumor suppressors
UCEC: Uterine cancer
VUS: Variant of unknown significance
