# Supplementary material for: Exome-wide analysis of bi-allelic alterations identifies a Lynch phenotype in The Cancer Genome Atlas
Source: Genome Med. 2018 Sep 14;10:69. doi: 10.1186/s13073-018-0579-5 (PMC6138910; doi:10.1186/s13073-018-0579-5)
Supplement: Supplementary file 1 — Figure S1. Calling somatic methylation status. Figure S2. Example LOH events. Figure S3. Genes frequently affected by germline:somatic alteration. Figure S4. Association between germline LOF burden and cancer type. Figure S5. Both germline and somatic LOF mutations can alter the same position. Figure S6. Mutational signature analysis of germline:somatic MMR alteration carriers. Figure S7. Mon-allelic germline variation in MMR pathway not associated with somatic MSI. Figure S8. Association testing between germline, somatic, and epigenetic alteration and somatic MSI burden. Figure S9. SHPRH methylation in uterine cancer. Figure S10. SHPRH expression in normal tissues. Figure S11. Mutational signature analysis of MLH1 and SHPRH methylated samples. Figure S12. Co-occurrence testing for SHPRH methylation. Figure S13. Mutational signature analysis of BRCA1/2 germline variant carriers. Figure S14. Mutational signature analysis of mono- and bi-allelic alteration of DDR pathways. Figure S15. Association between damaging germline variants and age of diagnosis. Table S6. Association between age of diagnosis and MMR pathway alteration. Table S7. ClinVar annotations for germline variants pathogenic for Lynch syndrome. Table S8. ClinVar annotations for germline variants of unknown significance. Table S9. Modeling a gene-level germline:somatic interaction for L-MMR genes. Table S10. Association between type of germline:somatic mutation and somatic MSI burden. Table S11. Association between germline:somatic mutation types and age of diagnosis. Table S12. Association between mono-allelic germline MMR variants and somatic MSI burden. Table S13. MSI linear model results using unfiltered somatic mutations and with germline:somatic MMR alteration carriers included. Table S14. Modeling somatic MSI burden using MMR perturbations highly correlated with SHPRH methylation. Table S15. Modeling somatic MSI burden using SHPRH expression. Table S17. Association between MMR, BRCA1/2, SDHB/RET, an [file 13073_2018_579_MOESM1_ESM.pdf]

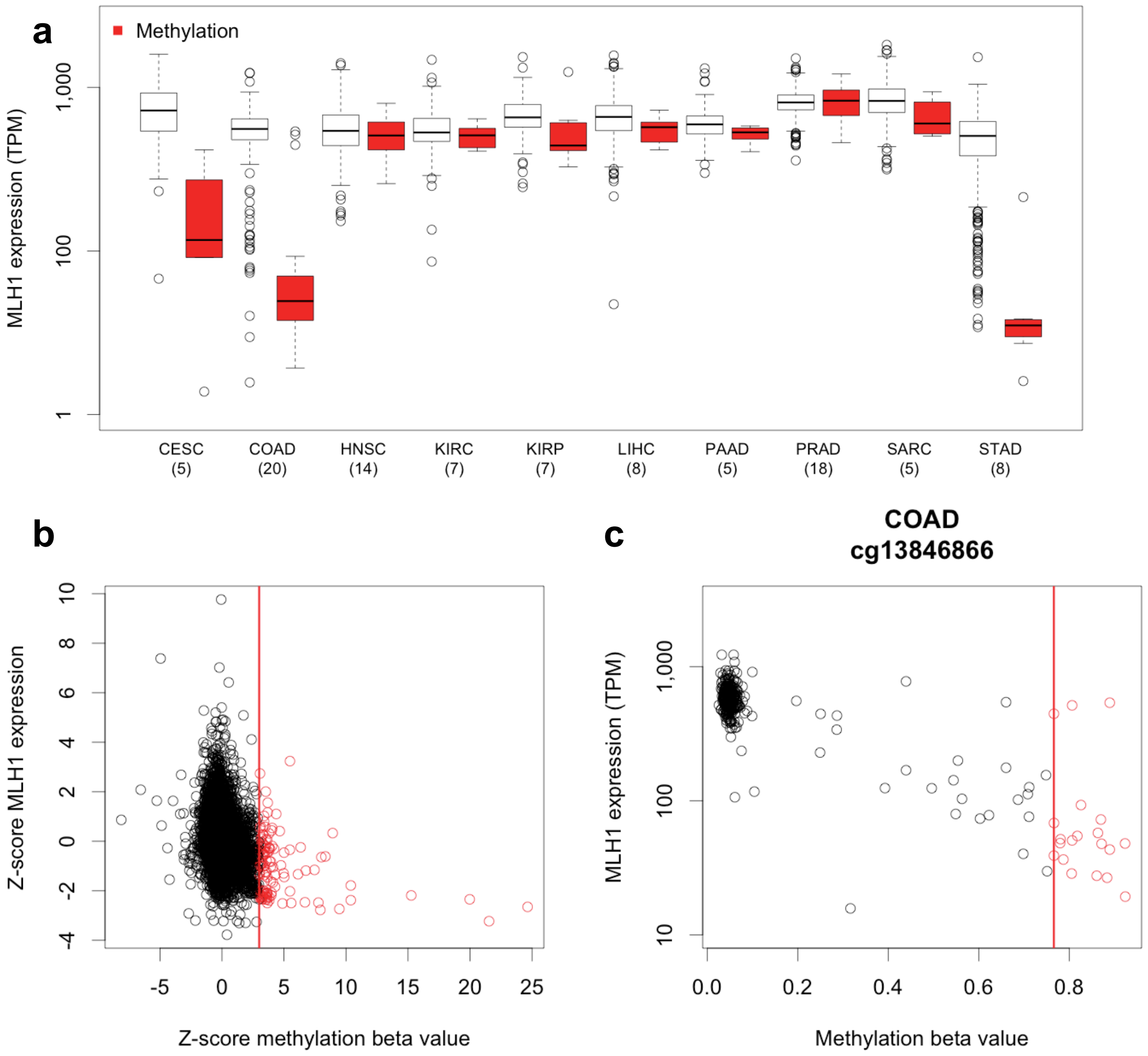

Figure S1. Calling somatic methylation status.
(a) *MLH1* expression is decreased in methylated samples. Cancer types with 5+ methylated samples are shown. TPM = transcripts per million. (b) Expression of *MLH1* vs. methylation beta value. Both expression values (in transcripts per million) and methylation beta values were converted to Z-scores using the mean and standard deviation for each cancer type. The red line indicates the cutoff used to call methylation. (c) Expression of *MLH1* vs. methylation beta value in colon cancer samples only. Beta values are from the methylation probe cg13846866, which was most anti-correlated with *MLH1* expression in colon cancer. The red line indicates the cutoff used to call methylation.

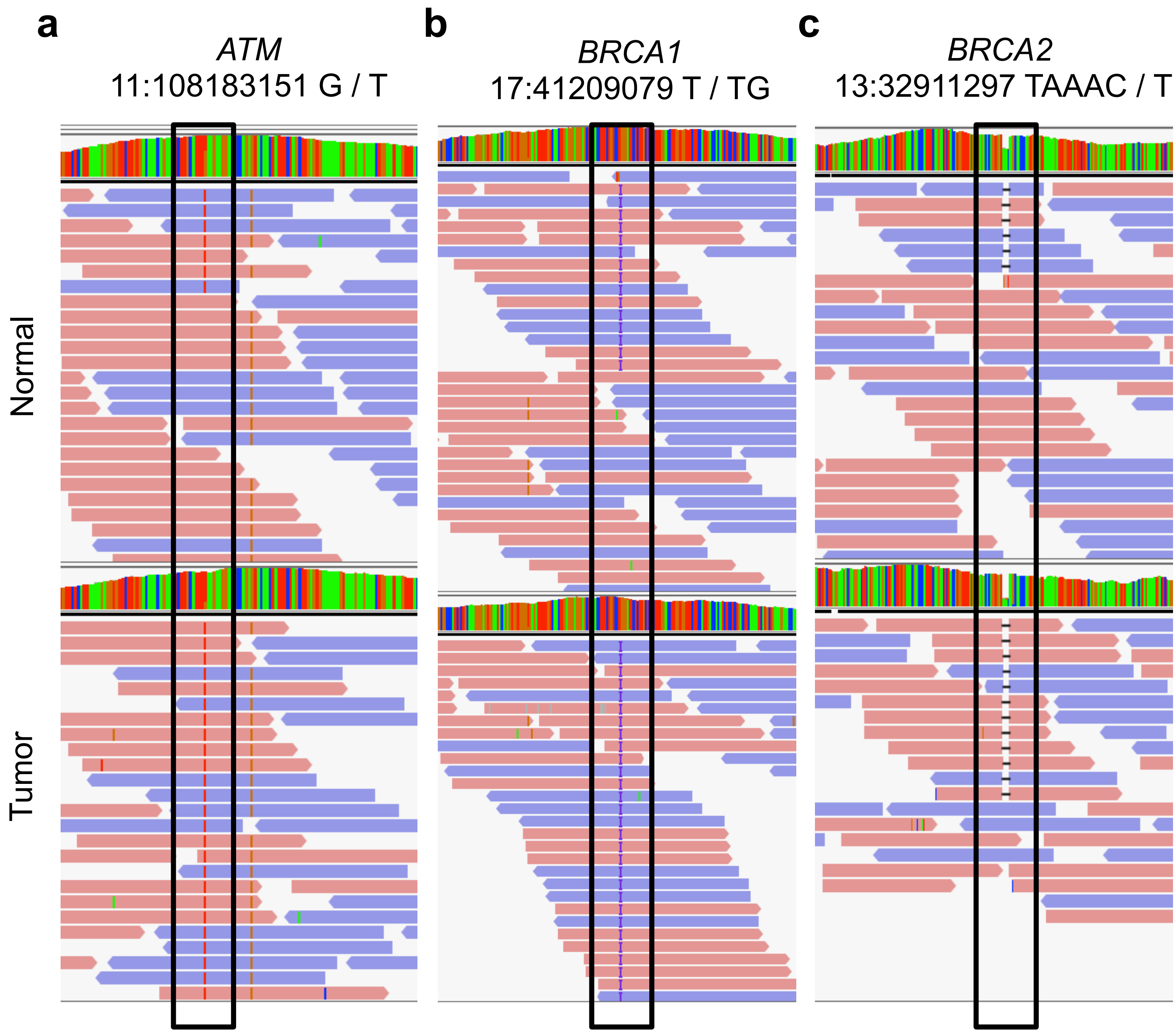

Figure S2. Example LOH events.
IGV snapshots of paired tumor:normal BAM files with a somatic LOH event for *ATM* (a), *BRCA1* (b), and *BRCA2* (c). The germline locus subject to LOH is highlighted with a box. Screenshots were taken with downsampling settings of max read count 20 per 200 base window.

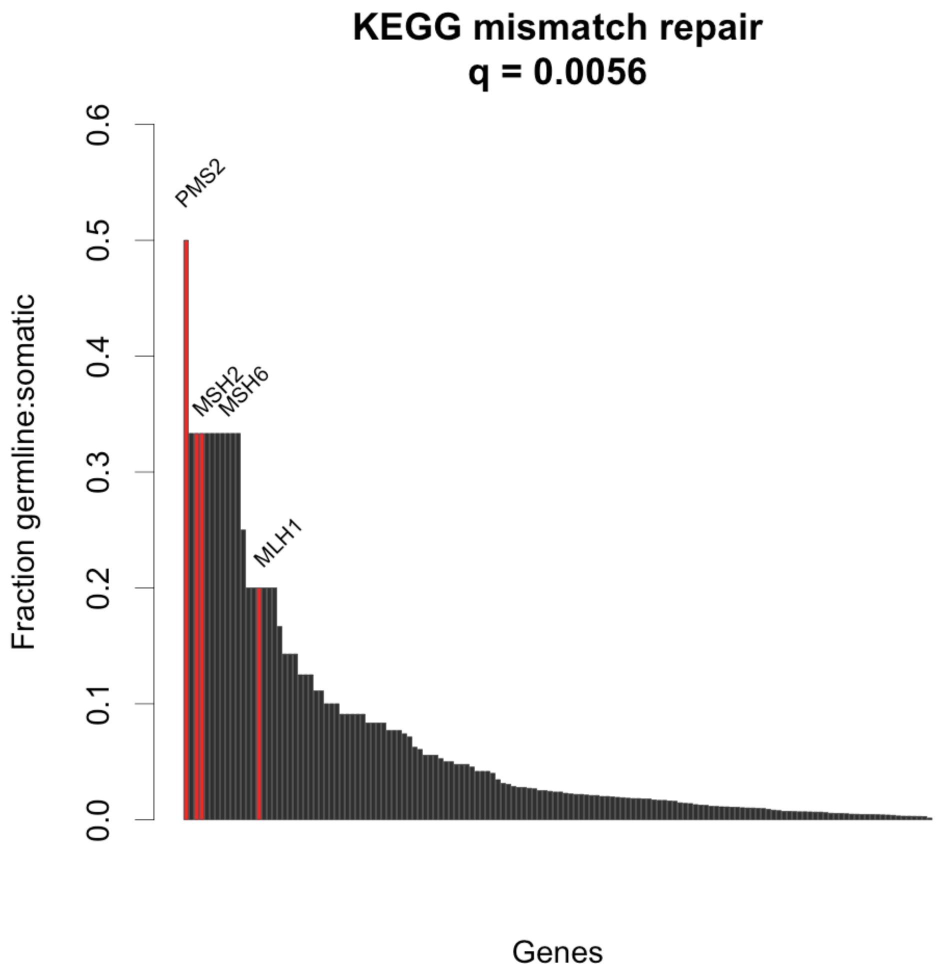

Figure S3. Genes frequently affected by germline:somatic alteration.
Barplot showing gene level frequency of germline:somatic alteration with KEGG mismatch repair pathway genes highlighted in red. Fraction germline:somatic alteration was calculated for each gene as number of germline:somatic alterations/number of germline LOF variants. Only genes with > 2 germline LOF variants in the cohort were included in the analysis. Significance was calculated using fgsea and is adjusted for multiple hypothesis testing[1].

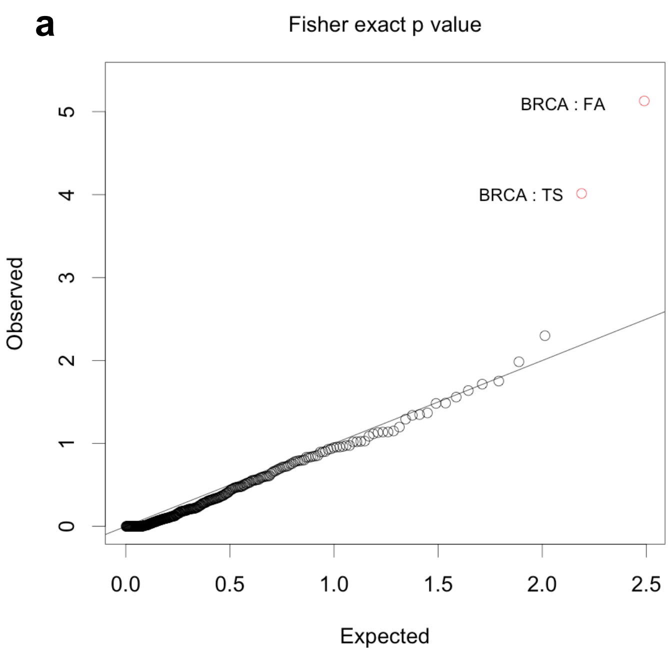

Figure S4. Association between germline LOF burden and cancer type.
QQ plot of Fisher exact p-values of the association between 11 DDR pathways and 28 cancer types using LOF variants only (a) or LOF and ClinVar pathogenic variants (b). Only samples determined to be of European descent using PCA were used (*n* = 7,734). Red indicates significance above a Bonferroni threshold. (a) Significant hits: breast cancer:tumor suppressor pathway ($p = 9.69e^{-5}$), breast cancer:Fanconi anemia pathway ($p = 7.42e^{-6}$). (b) Significant hits: pheochromocytoma and paraganglioma:cancer predisposition genes ($p = 1.18e^{-9}$), breast cancer:Fanconi anemia pathway ($p = 3.89e^{-7}$), pheochromocytoma and paraganglioma:oncogenes ($p = 2.30e^{-6}$).

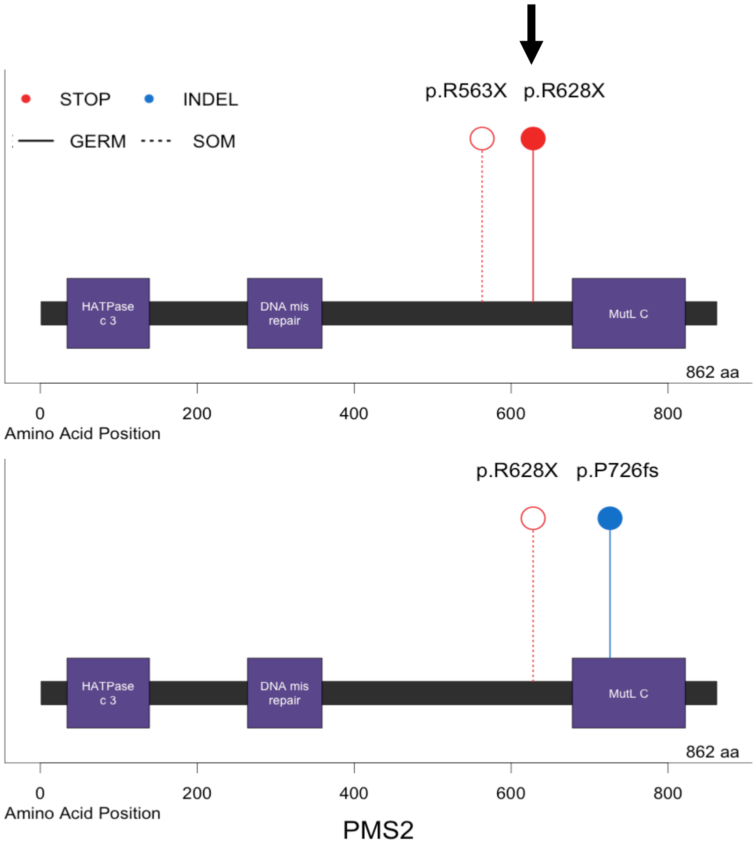

Figure S5. Both germline and somatic LOF mutations can alter the same position. Lollipop plot indicating the amino acids altered in two samples with *PMS2* bi-allelic alteration. Germline LOF variants are represented by solid lines, somatic LOF mutations by dashed lines. The stopgain mutation highlighted by an arrow, p.R628X, is inherited in one individual and acquired in the other. PFAM domain abbreviations: HATPase c 3 = Histidine kinase, DNA gyrase B, and HSP90-like ATPase; MutL C = MutL C terminal dimerization domain.

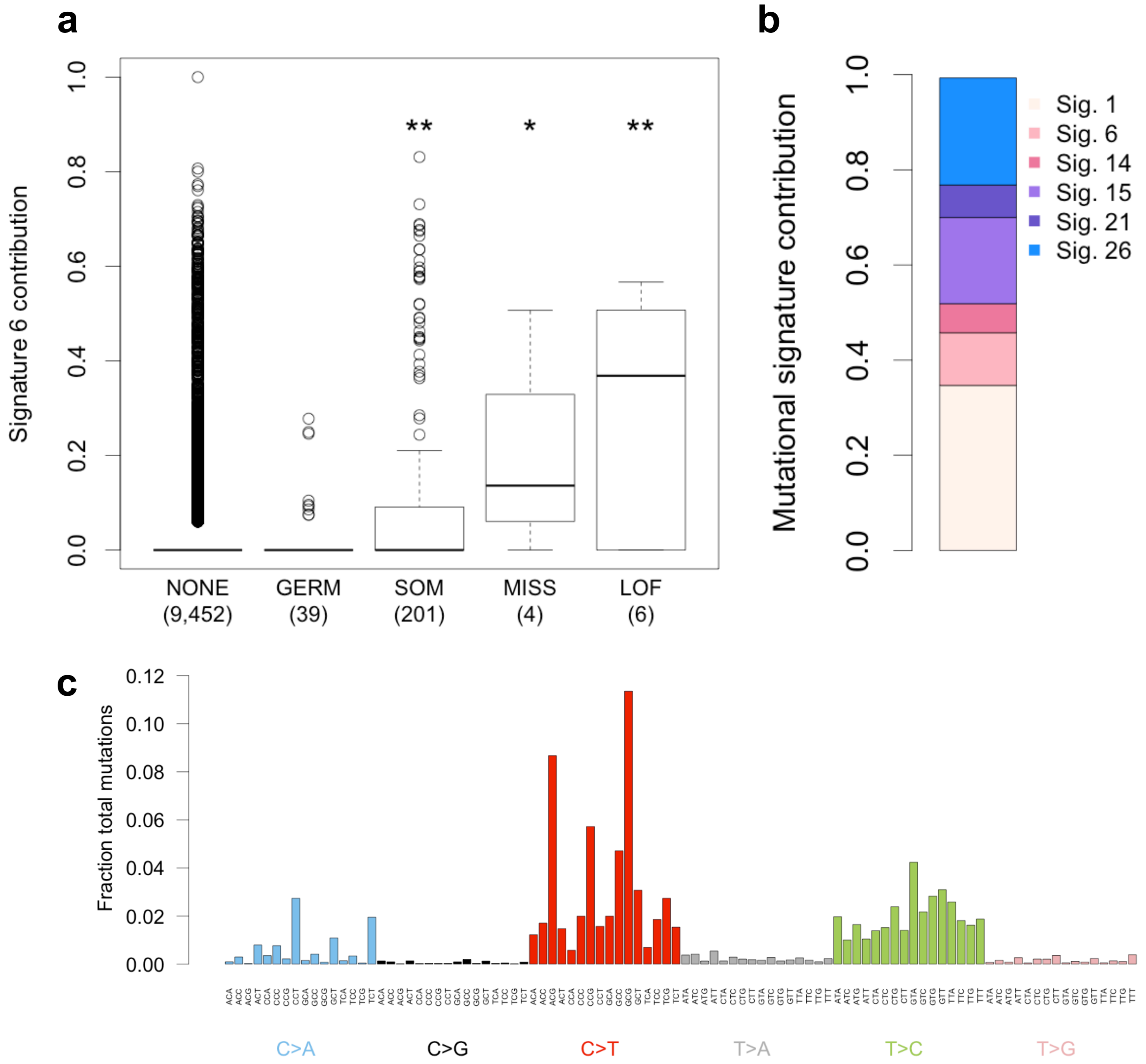

Figure S6. Mutational signature analysis of germline:somatic MMR alteration carriers. (a) Fraction of mutations attributed to mutational signature 6 plotted by type of germline:somatic MMR alteration. Individuals were grouped by MMR gene mutation type: NONE, no alteration; GERM, germline LOF variants only; SOM, somatic LOF mutations only; MISS, bi-allelic alteration including a missense mutation; LOF, bi-allelic alteration via dual LOF mutation. Wilcox p = 0.00023, 0.0063, and 0.00096; permutation p = 0.0002, 0.011, and 0.002 for SOM, MISS, and LOF respectively. (b) Fraction of combined germline:somatic alteration carriers mutational profile attributed to the mutational signatures available in COSMIC. (c) Mutational profile of all germline:somatic alteration carriers combined.

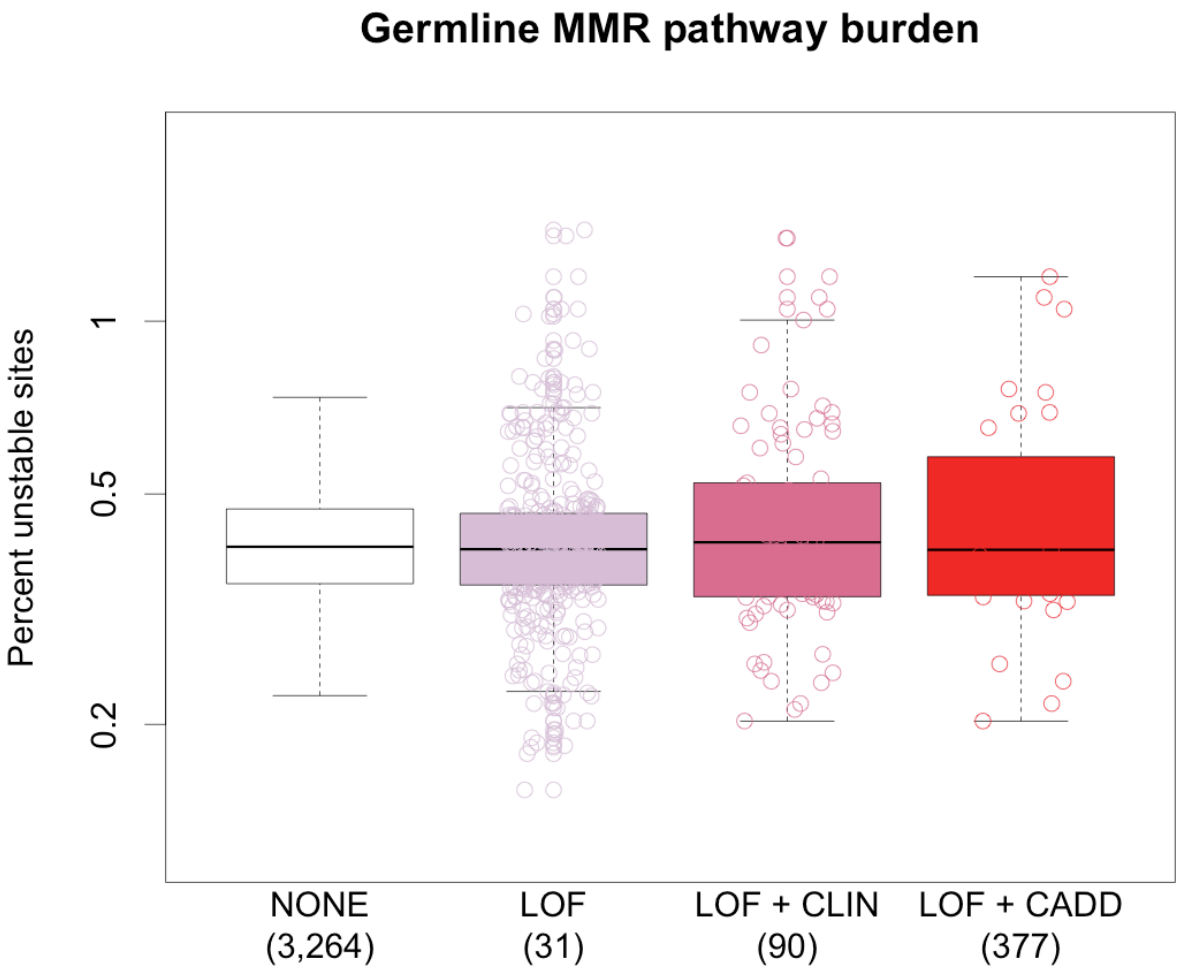

Figure S7. Mon-allelic germline variation in MMR pathway not associated with somatic MSI. Somatic MSI burden for individuals with MMR germline variants but no somatic alteration of the MMR pathway. NONE = no alteration of the MMR pathway, LOF = germline LOF variant in an MMR gene, LOF + CLIN = germline LOF variant or ClinVar pathogenic variant in an MMR gene, LOF + CADD = germline LOF variant or variant with a CADD score ≥ 30 in an MMR gene. The number of samples in each category is displayed in parentheses.

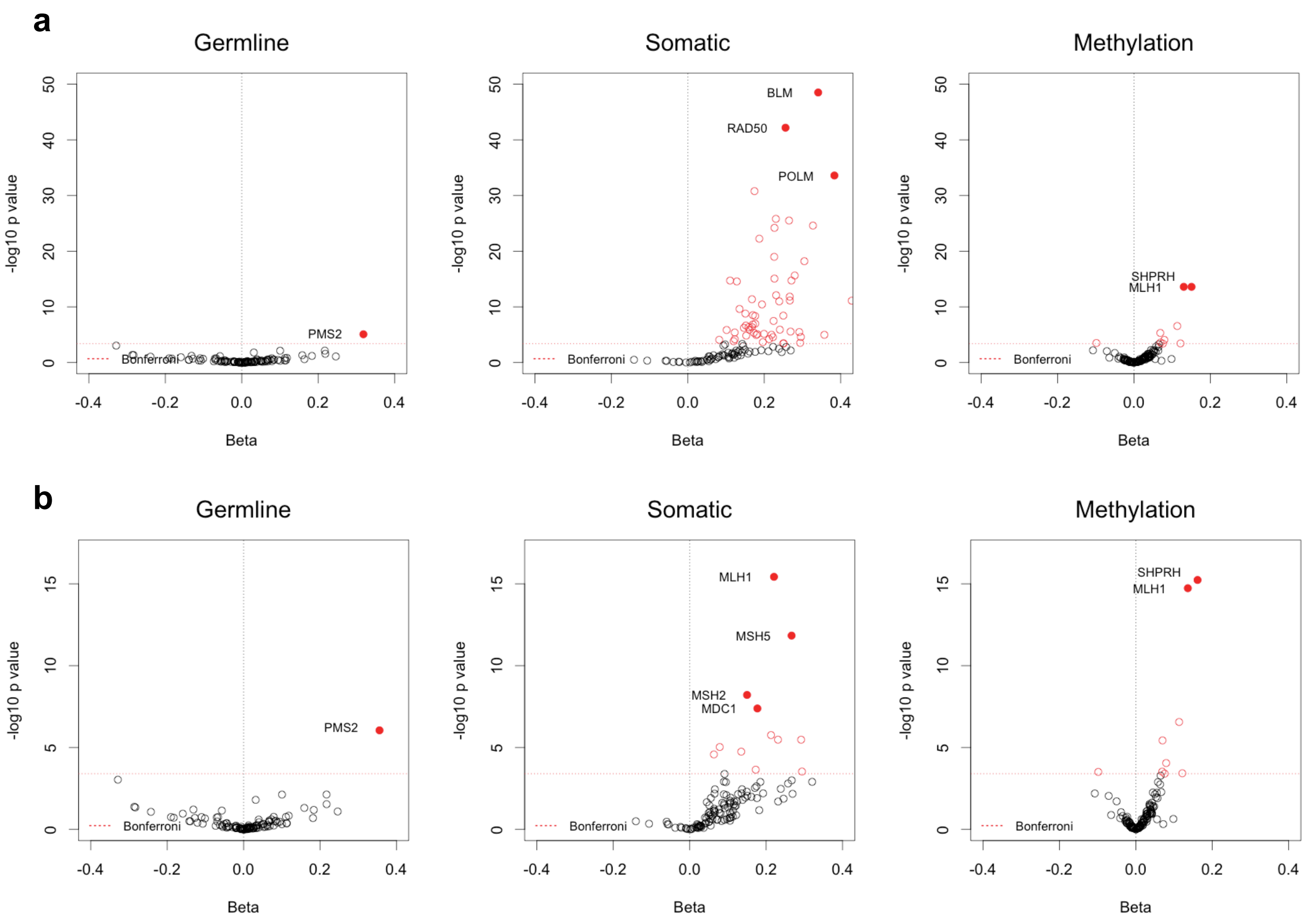

Figure S8. Association testing between germline, somatic, and epigenetic alteration and somatic MSI burden.

(a) The same analysis as Fig. 4, but including all somatic LOF mutations. (b) The same analysis as Fig. 4, but including germline:somatic MMR alteration carriers.

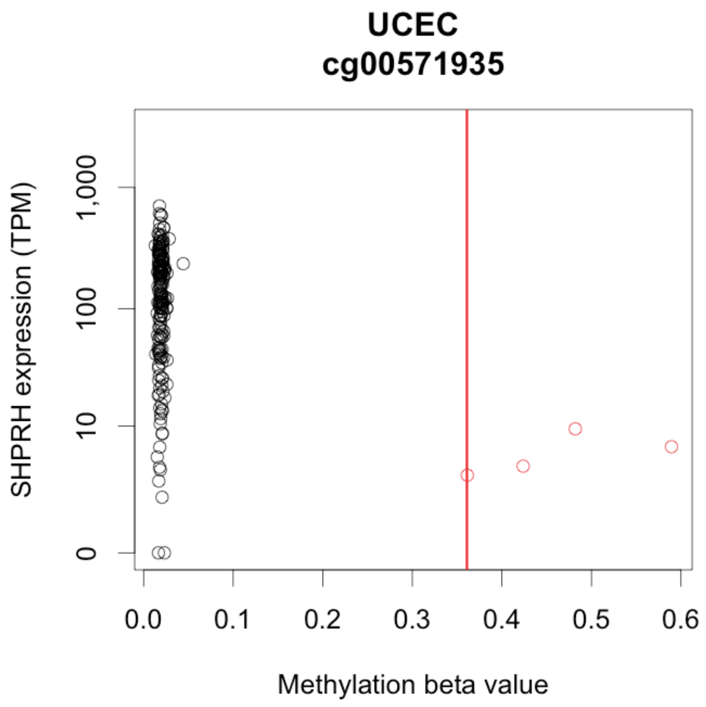

Figure S9. *SHPRH* methylation in uterine cancer.
Expression of *SHPRH* vs. methylation beta value in uterine cancer samples only. Beta values are from the methylation probe cg00571935, which was most anti-correlated with *SHPRH* expression in uterine cancer. The red line indicates the cutoff used to call methylation.

a

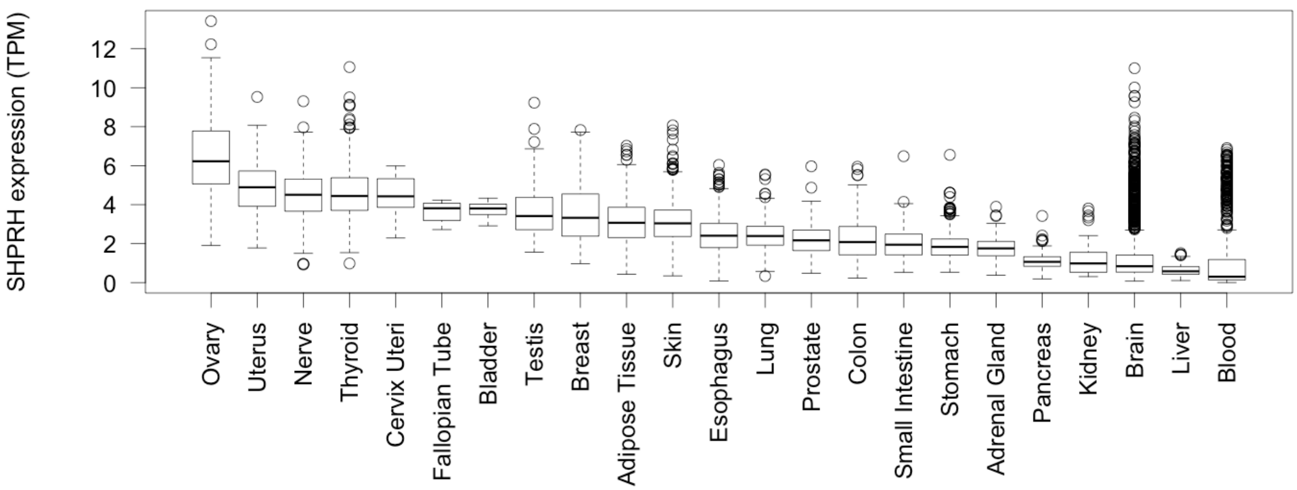

b

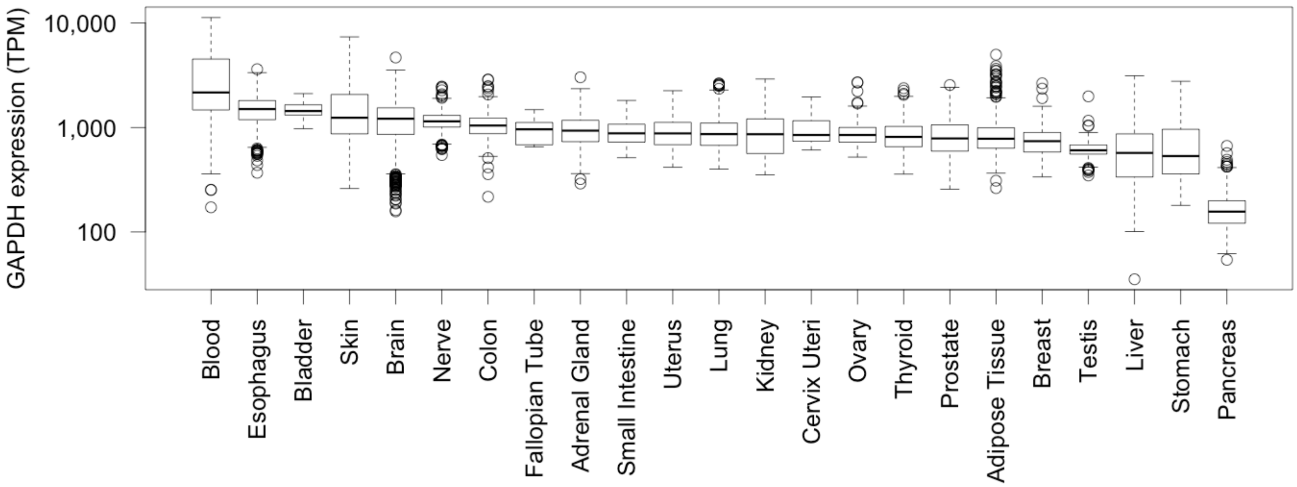

Figure S10. *SHPRH* expression in normal tissues.
Expression of *SHPRH* (a) or *GAPDH* (b) in 23 normal tissue types represented in GTEx. TPM = transcripts per million.

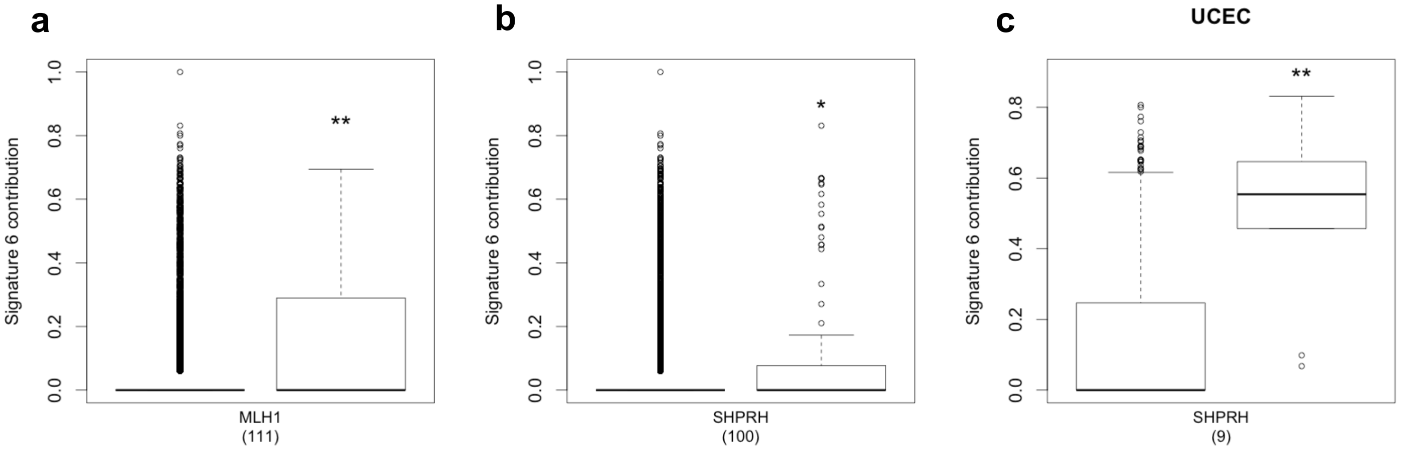

Figure S11. Mutational signature analysis of *MLH1* and *SHPRH* methylated samples.
(a) Fraction of mutations attributed to mutational signature 6 plotted by *MLH1* methylation status. Wilcox p = $3.882e^{-15}$, permutation p < $1e^{-4}$. (b) Fraction of mutations attributed to mutational signature 6 plotted by *SHPRH* methylation status. Wilcox p = 0.041, permutation p = 0.0264. (c) Fraction of mutations attributed to mutational signature 6 plotted by *SHPRH* methylation status in uterine cancer samples only. Wilcox p = $3.405e^{-5}$, permutation p < $1e^{-4}$.

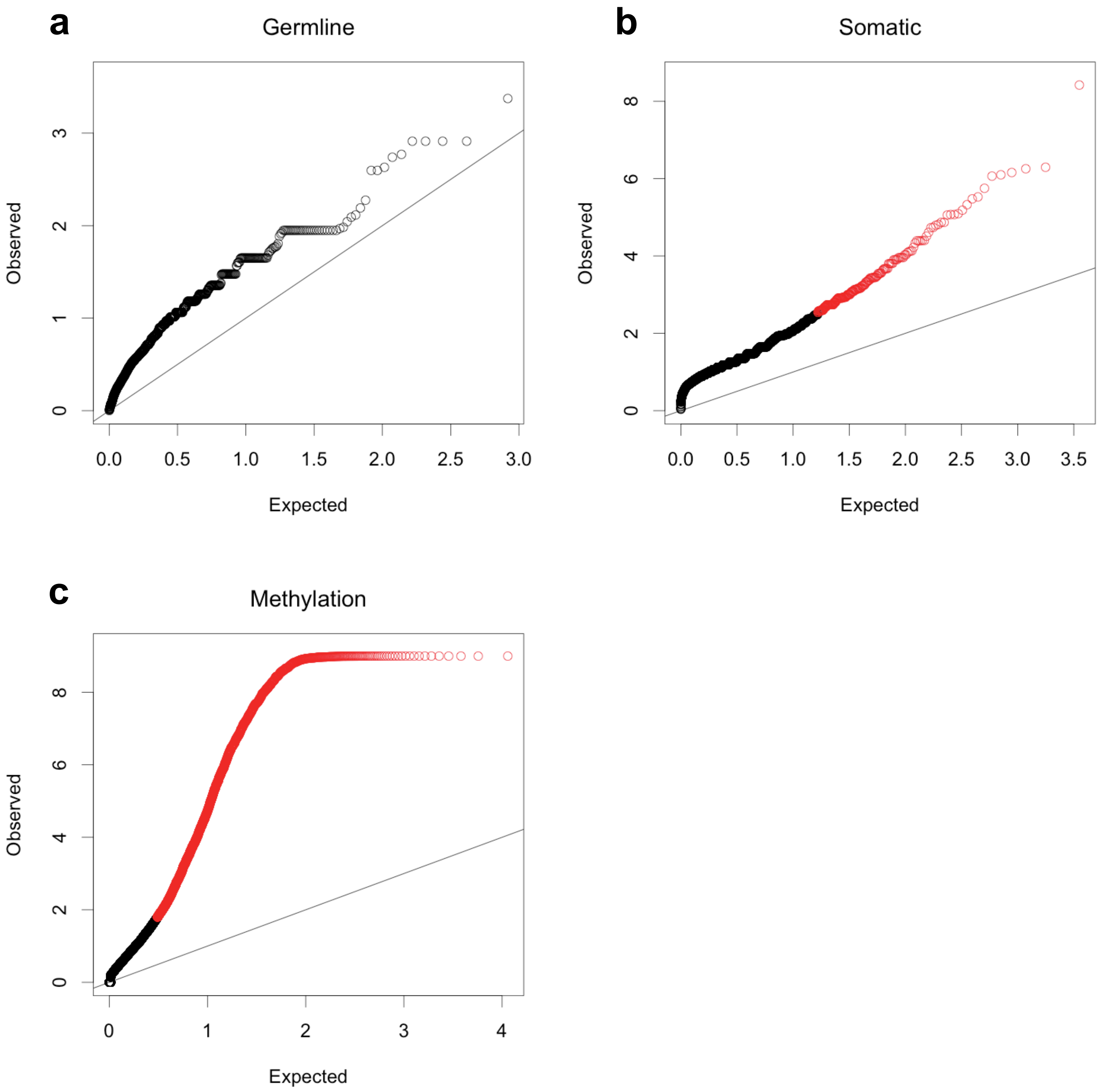

Figure S12. Co-occurrence testing for *SHPRH* methylation.
QQ plots of co-occurrence testing between *SHPRH* methylation and germline LOF variants (a), somatic LOF mutations (b), and somatic methylation (c) in 9,484 genes and n = 8,087 samples. Red indicates 5% FDR. Somatic LOF mutations in 213 genes and somatic methylation of 3,694 genes significantly co-occur with *SHPRH* methylation. Of these co-occurring genes, 8 are MMR pathway genes (*MLH3, PCNA, POLD3, RFC1, RFC5, RPA3, MLH3*, and *PMS2*).

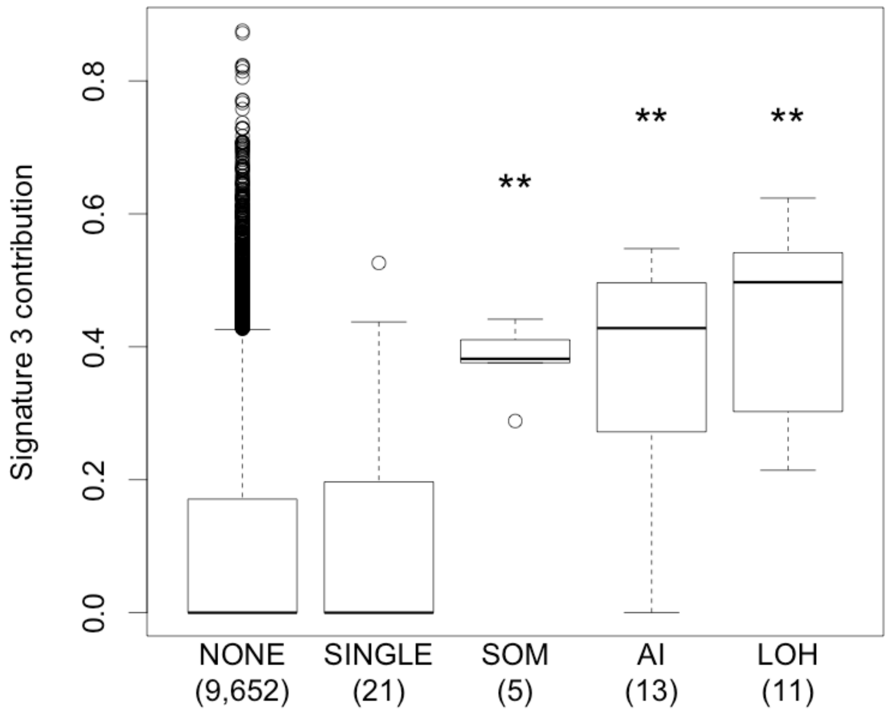

Figure S13. Mutational signature analysis of *BRCA1/2* germline variant carriers.
Fraction of mutations attributed to mutational signature 3 plotted by *BRCA1/2* status. Single = germline LOF or ClinVar pathogenic variant only, SOM = bi-allelic alteration via somatic mutation, AI = allelic imbalance, LOH = loss of heterozygosity. Wilcoxon rank sum test p = $2.246e^{-4}$, $6.981e^{-6}$, $2.343e^{-7}$; permutation p $< 1e^{-4}$, for SOM, AI, and LOH respectively.

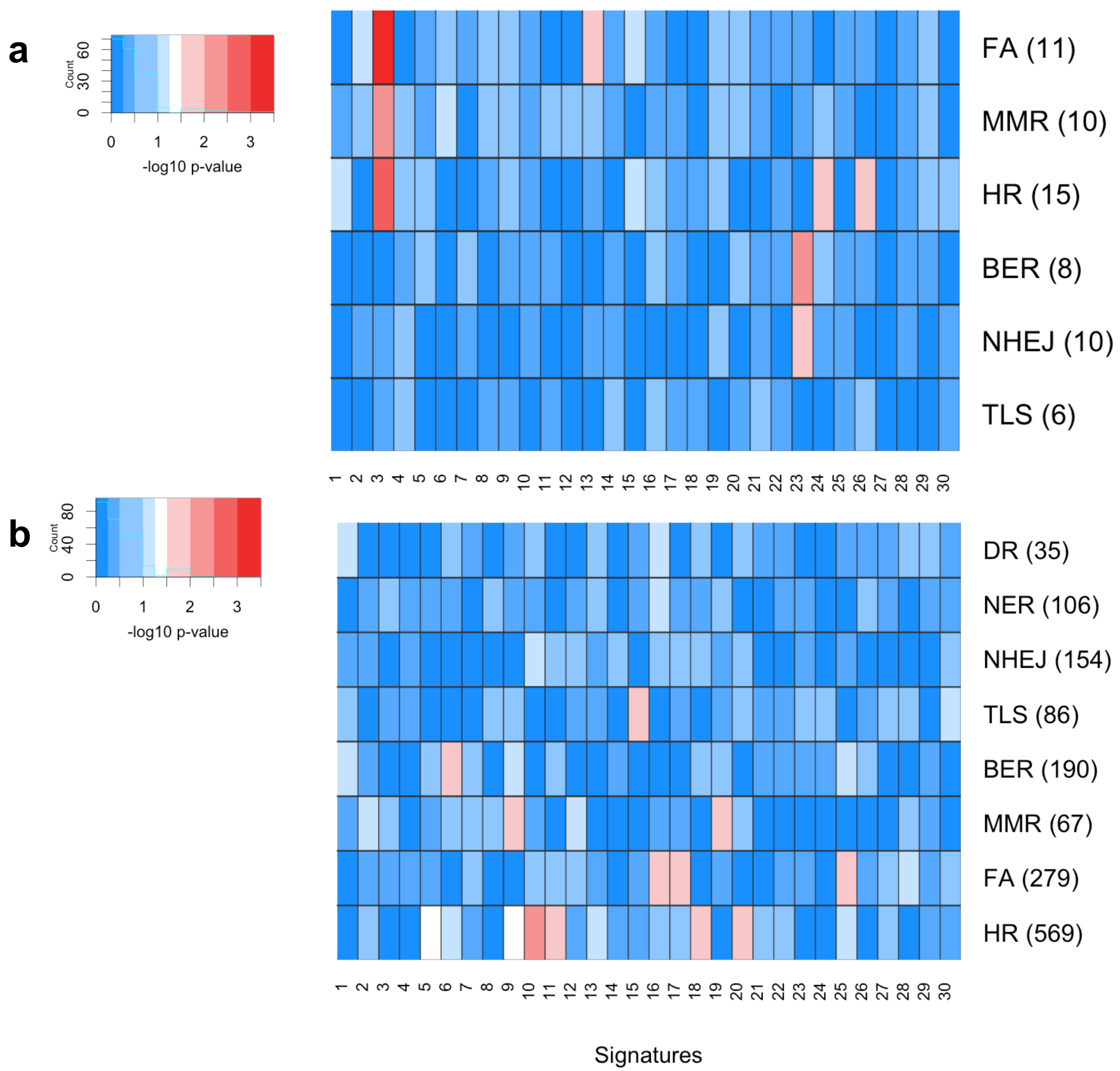

Figure S14. Mutational signature analysis of mono- and bi-allelic alteration of DDR pathways. A Wilcoxon rank sum test was used to test for differences in somatic mutational signature burden between individuals carrying DDR alterations vs. those without. (a) Heatmap of p values for the association between bi-allelic alteration in 6 DDR pathways and 30 mutational signatures in COSMIC. (b) Heatmap of p values for the association between mono-allelic alteration in 8 DDR pathways and 30 mutational signatures in COSMIC.

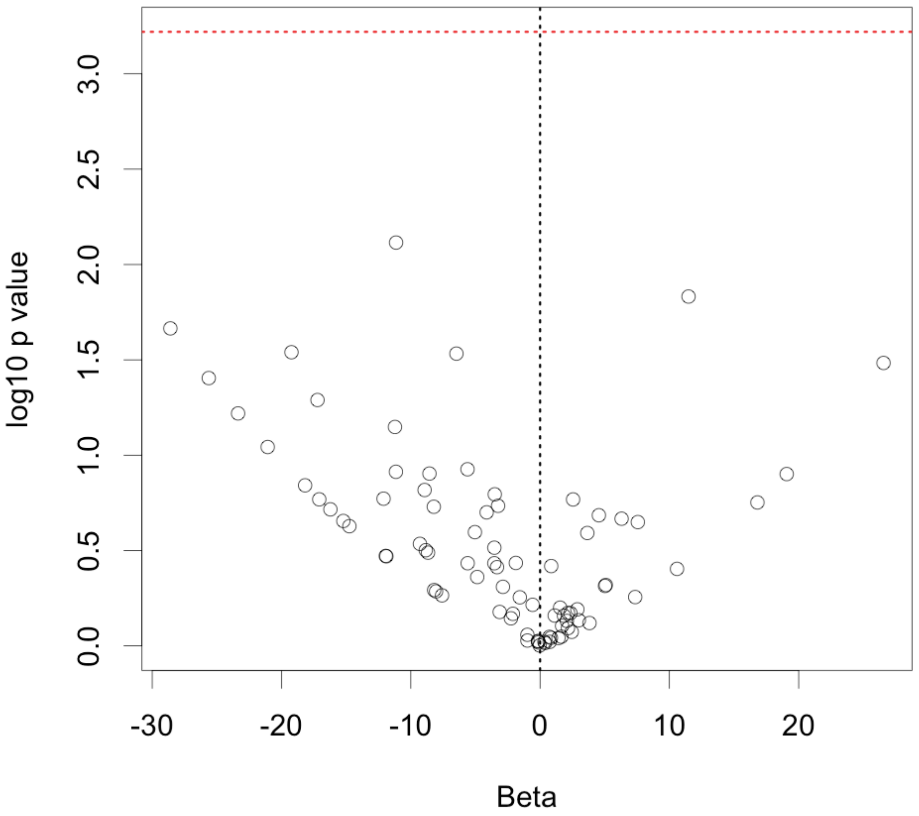

Figure S15. Association between damaging germline variants and age of diagnosis. Volcano plot of association testing between germline LOF and pathogenic ClinVar variant carrier status and age of diagnosis for 85 cancer predisposition genes in *n* = 8,913 samples.

Table S5. Association between somatic MSI burden and MMR pathway alteration.

	p value	Beta	Std. Error	t value
Germ	0.756	0.007	0.024	0.310
Som	$1.53e^{-20}$	0.104	0.011	9.331
Meth	$5.44e^{-05}$	0.022	0.005	4.039
Mixed germ	0.0382	0.116	0.056	2.073
Mixed	$2.33e^{-14}$	0.167	0.021	7.653
B-allelic Som	0.574	0.044	0.079	0.561
Germline:Methylation	0.285	0.146	0.137	1.068
Germline:Somatic	$3.95e^{-15}$	0.444	0.056	7.881

Table S6. Association between age of diagnosis and MMR pathway alteration.

	p value	Beta	Std. Error	t value
Germ	0.448	-2.251	2.967	-0.758
Som	0.698	-0.536	1.384	-0.387
Meth	0.226	0.831	0.686	1.211
Mixed germ	0.082	12.040	6.935	1.736
Mixed	0.827	-0.590	2.703	-0.218
B-allelic Som	0.921	-0.975	9.832	-0.099
Germline:Methylation	0.861	2.966	16.981	0.174
Germline:Somatic	0.0418	-14.164	6.957	-2.035

Table S7. ClinVar annotations for germline variants pathogenic for Lynch syndrome.

Source	Type	Chr	Pos	Ref	Alt	Gene	TX	AA change	ExAC AF	ClinVar Significance	Num. Submissions
TCGA	LOF	3	37053589	C	T	*MLH1*	NM_000249	p.R226X	singleton	Pathogenic	8
TCGA	LOF	2	47703538	C	T	*MSH2*	NM_000251	p.R680X	singleton	Pathogenic	10
TCGA	LOF	7	6026709	G	A	*PMS2*	NM_000535	p.R563X	1.65E-05	Pathogenic	4
TCGA	LOF	7	6026514	G	A	*PMS2*	NM_000535	p.R628X	3.30E-05	Pathogenic	5
TCGA	MISS	2	47693947	G	A	*MSH2*	NM_000251	p.S554N	singleton	Pathogenic	1

Table S8. ClinVar annotations for germline variants of unknown significance.

Source	Type	Chr	Pos	Ref	Alt	Gene	TX	AA change	ExAC AF	ClinVar Significance	Num. Submissions
TCGA	LOF	2	48027685	AT	A	*MSH6*	NM_000179	p.I855fs	singleton	NA	NA
ClinVar	LOF	2	48027690	TGATT	T	*MSH6*	NM_000179	p.D857fs	NA	Pathogenic	2
TCGA	MISS	2	48026417	T	G	*MSH6*	NM_0001792	p.F432C	singleton	NA	NA
ClinVar	MISS	2	48026417	T	C	*MSH6*	NM_0001792	p.F432S	NA	Pathogenic	4
ClinVar	MISS	2	48026418	T	G	*MSH6*	NM_0001792	p.F432L	NA	Pathogenic	2
TCGA	LOF	3	37092010	A	T	*MLH1*	NM_000249	p.K713X	singleton	NA	NA
ClinVar	LOF	3	37092009	G	A	*MLH1*	NM_000249	p.W712X	NA	Pathogenic	1
TCGA	MISS	2	47702251	C	G	*MSH2*	NM_000251	p.P616R	4.12E-05	VUS	5

Table S9. Modeling a gene-level germline:somatic interaction for L-MMR genes.

Gene	Alteration	p value	Beta	Std. Error	t value
MLH1	Germ	0.043	0.197	0.097	2.018
MLH1	Som	$3.54e^{-16}$	0.223	0.027	8.180
MLH1	Meth	$1.91e^{-15}$	0.136	0.017	7.972
MLH1	Germ * Som	0.488	-0.119	0.171	-0.693
MSH2	Germ	0.201	0.181	0.142	1.276
MSH2	Som	$6.10e^{-09}$	0.15006	0.025	5.824
MSH2	Meth	0.036	0.0443	0.021	2.094
MSH2	Germ * Som	NA	NA	NA	NA
MSH5	Germ	0.138	-0.0689	0.046	-1.482
MSH5	Som	$1.71e^{-09}$	0.234	0.038	6.034
MSH5	Meth	0.019	-0.0501	0.021	-2.345
MSH5	Germ * Som	$9.29e^{-4}$	0.502	0.151	3.312
MSH6	Germ	0.523	0.0896	0.140	0.638
MSH6	Som	0.019	0.0401	0.030	1.300
MSH6	Meth	0.161	0.0289	0.020	1.400
MSH6	Germ * Som	0.990	-0.00250	0.200	-0.012
PMS2	Germ	0.014	0.241	0.098	2.44
PMS2	Som	$03.33e^{-3}$	0.0797	0.037	2.128
PMS2	Meth	0.841	0.00514	0.025	0.200
PMS2	Germ * Som	0.091	0.2440	0.1445	1.688

Table S10. Association between type of germline:somatic mutation and somatic MSI burden.

MMR Category	p value	Beta	Std. Error	t value
NONE	$5.13e^{-05}$	-0.0224	0.005	-4.053
GERM	0.738	-0.007	0.022	-0.335
SOM	$5.70e^{-16}$	0.089	0.011	8.122
MISS	$1.01e^{-10}$	0.446	0.068	6.479
LOF	$2.78e^{-15}$	0.445	0.056	7.925

Table S11. Association between germline:somatic mutation types and age of diagnosis.

MMR Category	p value	Beta	Std. Error	t value
NONE	0.250	-0.388	0.337	-1.149
GERM	0.315	-2.018	2.008	-1.004
SOM	0.151	-1.34	0.934	-1.435
MISS	0.949	-0.398	6.232	-0.063
LOF	$1.12e^{-03}$	-16.603	5.093	-3.259

Table S12. Association between mono-allelic germline MMR variants and somatic MSI burden.

	LOF				CLINVAR + LOF				CADD 30 + LOF			
	p value	Beta	Std. Error	t value	p value	Beta	Std. Error	t value	p value	Beta	Std. Error	t value
GERM	0.337	0.021	0.021	0.959	0.125	0.020	0.013	1.913	0.831	0.001	0.006	0.212

Table S13. MSI linear model results using unfiltered somatic mutations and with germline:somatic MMR alteration carriers included.

Perturbation	Gene	All Samples, MSI filter				All Samples, no MSI filter				Lynch-like samples removed, MSI filter			
		p value	Beta	Std. Error	t value	p value	Beta	Std. Error	t value	p value	Beta	Std. Error	t value
Germ	*PMS2*	$8.82e^{-07}$	0.355	0.072	4.922	$8.19e^{-06}$	0.318	0.071	4.464	0.830	-0.029	0.138	-0.214
Som	*MDC1*	$4.08e^{-08}$	0.177	0.032	5.495	$3.61e^{-11}$	0.194	0.029	6.634	$7.13e^{-05}$	0.133	0.033	3.973
Som	*MSH2*	$6.10e^{-09}$	0.150	0.025	5.824	$4.22e^{-12}$	0.167	0.024	6.946	$2.77e^{-07}$	0.133	0.026	4.963
Som	*MSH5*	$1.45e^{-12}$	0.266	0.037	7.097	$1.45e^{-12}$	0.266	0.037	7.097	$7.93e^{-10}$	0.236	0.038	6.157
Som	*MLH1*	$3.73e^{-16}$	0.220	0.026	8.174	$1.02e^{-19}$	0.226	0.024	9.125	$6.29e^{-17}$	0.226	0.027	8.391
Meth	*MLH1*	$1.85e^{-15}$	0.136	0.017	7.976	$2.49e^{-14}$	0.130	0.017	7.645	$6.60e^{-16}$	0.136	0.016	8.101
Meth	*SHPRH*	$5.82e^{-16}$	0.161	0.019	8.120	$2.50e^{-14}$	0.15	0.019	7.644	$1.19e^{-16}$	0.163	0.019	8.309

Table S14. Modeling somatic MSI burden using MMR perturbations highly correlated with *SHPRH* methylation.

Perturbation	Gene	p value	Beta	Std. Error	t value
Meth	*MLH3*	0.949	-0.001	0.019	-0.064
Meth	*PCNA*	0.0447	0.036	0.018	2.007
Meth	*POLD3*	0.0400	-0.069	0.033	-2.054
Meth	*RFC1*	0.223	0.027	0.022	1.218
Meth	*RFC5*	0.339	0.016	0.016	0.956
Meth	*RPA3*	0.155	0.025	0.017	1.423
Meth	*SHPRH*	$3.61e^{-16}$	0.164	0.020	8.180
Som	*PMS2*	0.0101	0.098	0.038	2.574
Som	*MLH3*	0.0570	0.060	0.031	1.903

Table S15. Modeling somatic MSI burden using *SHPRH* expression.

	p value	Beta	Std. Error	t value
SHPRH expression	0.000152	$-4.487e^{-05}$	$1.184e^{-05}$	-3.791

Table S17. Association between MMR, *BRCA1/2*, *SDHB/RET*, and *TP53* germline variant carrier status and age of diagnosis.

Gene Set	Cancer Type	p value	Beta	Std. Error	t value
MMR	Expected	0.011	-9.995	3.950	-2.530
BRCA1/2	Expected	0.030	-5.939	2.745	-2.163
BRCA1/2 NON	Other	0.455	-1.697	2.275	-0.746
TP53 CANCERTYPE	Expected	0.073	-6.198	3.466	-1.788
TP53 NON	Other	0.513	-1.920	2.935	-0.654
SDHB/RET CANCERTYPE	Expected	0.012	-7.738	3.085	-2.508
SDHB/RET NON	Other	0.780	-1.096	3.934	-0.279

Table S18. Association between predisposition gene germline variant carrier status and age of diagnosis.

Gene Set	p value	Beta	Std. Error	t value
Other Predisposition	0.271	-0.541	0.492	-1.099
Known	$4.52e^{-05}$	-4.710	1.154	-4.081
